# Acetate‐Linked Energy Metabolism as a Determinant of Early *Haemophilus influenzae* Infection Fitness

**DOI:** 10.1002/mbo3.70324

**Published:** 2026-06-14

**Authors:** Marufa Nasreen, Jennifer Hosmer, Saurab Kishore Munshi, Riya Joshi, Ayaho Yamamoto, Horst J. Schirra, Peter Sly, Alastair G. McEwan, Ulrike Kappler

**Affiliations:** ^1^ School of Chemistry and Molecular Biosciences The University of Queensland St. Lucia Queensland Australia; ^2^ Child Health Research Centre The University of Queensland South Brisbane Queensland Australia; ^3^ School of Environment and Science, Griffith Institute for Drug Discovery Griffith University Nathan Queensland Australia; ^4^ Centre for Advanced Imaging The University of Queensland Brisbane Queensland Australia; ^5^ Institute for Biomedicine and Glycomics Griffith University Southport Queensland Australia

**Keywords:** acetate, *Haemophilus influenzae*, infectious diseases, metabolism, redox biology

## Abstract

Acetate is a major metabolic end‐product of *Haemophilus influenzae* and is produced via the phosphotransacetylase–acetate kinase (Pta–AckA) pathway, which also generates adenosine triphosphate (ATP). Despite its importance, the contribution of this pathway to *H. influenzae* physiology and virulence remains poorly understood. Here, we have investigated the individual roles of AckA and Pta for these processes by generating nonpolar single‐gene knockouts in a chronic obstructive pulmonary disease (COPD)‐isolate strain (Hi2019) and characterized their metabolic and infection phenotypes. Both mutants exhibited significant growth impairments under microaerobic and anaerobic conditions, with reductions in growth rate of up to 50% compared with the wild type. Loss of AckA or Pta decreased ATP levels to ~ 50% of wild‐type values and caused marked overoxidation of the NAD^+^/NADH pool. Metabolomic analyses revealed distinct perturbations at the pyruvate node: the *pta* mutant produced minimal acetate but accumulated pyruvate and d‐lactate, while the *ackA* mutant continued to produce acetate, likely via nonenzymatic breakdown of acetyl‐phosphate. Both mutations also increased sensitivity to oxidative stress and enhanced biofilm formation. In infection models, including bronchial epithelial cells, primary human nasal epithelia, and murine macrophages, intracellular survival of both mutants was significantly reduced during early infection stages, although attenuation diminished over time. These findings demonstrate that the Pta–AckA pathway is critical for metabolic homeostasis, stress resistance, and early intracellular colonization, highlighting its potential as a target for management of early‐stage *H. influenzae* infections.

## Graphical abstract

The Pta–AckA pathway is critical for *Haemophilus influenzae* energy generation, metabolic homeostasis, stress resistance, and intracellular colonization, highlighting its potential as a target for management of *H. influenzae* infections.

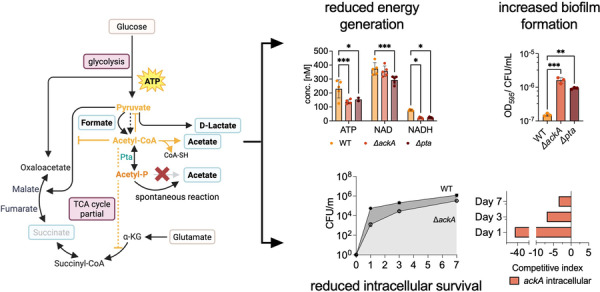

## 1 Introduction

Acetate, a short‐chain fatty acid (SCFA), is naturally produced by bacterial, fungal, and mammalian cells as an end product of fermentation processes (Bernal et al. 2016; Hosmer et al. 2024; Wolfe 2005). In the human body, acetate production is often driven by the microbiota, and the highest concentrations of acetate are found in the gut due to the activity of Bacteroidetes (Cummings et al. 1987). However, acetate production also occurs in other body niches, including the respiratory tract, where *Haemophilus influenzae* is a major acetate producer (Park et al. 2019; López‐López et al. 2020; Hosmer et al. 2022; Ramos‐Montañez et al. 2010; Sadykov et al. 2013). *H. influenzae* is a host‐adapted pathobiont that colonizes the human nasopharynx as a commensal, but can cause acute disease in both the upper and lower respiratory tract, and these infections can progress to invasive diseases, such as bacteraemia (Erwin and Smith 2007; Ahearn et al. 2017; Green et al. 2014; King 2012; Swords 2012; Duell et al. 2016; Essilfie et al. 2011; Langereis and de Jonge 2015; Van Eldere et al. 2014). *H. influenzae* strains are classified as either typeable or nontypeable based on the presence or absence, respectively, of a polysaccharide capsule (Langereis and de Jonge 2015). Currently, nontypeable *H. influenzae* (NTHi) strains account for 78% of invasive *H. influenzae* infections and are the most common type of clinical isolate (Ahearn et al. 2017; Whittaker et al. 2017). *H. influenzae* has a nutritional profile that is highly adapted to the respiratory tract and includes organic acids, such as lactate, pentose, and hexose sugars, as well as nucleosides (Hosmer et al. 2022). During aerobic growth on these substrates, acetate is produced almost exclusively as the main metabolic end‐product, while under oxygen limitation, formate and succinate are also produced. However, acetate always remains the major metabolic end‐product in *H. influenzae* (López‐López et al. 2020; Hosmer et al. 2022; Muda et al. 2019; Othman et al. 2014), as all *H. influenzae* strains have an incomplete tricarboxylic acid cycle (TCA). This incomplete TCA cycle contains only the reactions from α‐ketoglutarate dehydrogenase to malate dehydrogenase and consequently also lacks the reactions of the glyoxylate shunt (Othman et al. 2014; Tatusov et al. 1996). This unusual configuration means that acetyl‐CoA produced from pyruvate cannot be funnelled into the TCA cycle, and can either be used to produce acetate or routed towards fatty acid biosynthesis. Acetate production during energy metabolism is related to the co‐called “overflow metabolism,” where bacteria switch from energy generation by respiration to the less energy‐preserving production of acetate (de Groot et al. 2020; Vazquez and Oltvai 2016; Zeng and Yang 2019). In *H. influenzae*, the loss of a functional TCA cycle has essentially “locked” this bacterium into acetate production. While a respiratory chain is present, *H. influenzae* is incapable of respiratory oxidation of carbon substrates to CO2, and will always excrete partially oxidized carbon compounds as metabolic end‐products. We have previously described this type of metabolism as “respiration‐assisted fermentation,” as the respiratory chain can aid in redox balancing (Othman et al. 2014). The specific configuration of *H. influenzae* carbon metabolism makes acetate production a major pathway for energy generation under all growth conditions encountered.

A range of pathways mediate acetate production in bacteria, but the most common pathway uses a combination of the enzymes phosphotransacetylase (Pta) and acetate kinase (AckA), which is the case for facultative aerobes, including *H. influenzae* (Hosmer et al. 2024; Sadykov et al. 2013; Post et al. 2017; Zhang et al. 2021). The reaction catalyzed by Pta is reversible, and for acetate production, Pta converts acetyl‐CoA to acetyl‐phosphate (acetyl‐P), which is then converted to acetate and adenosine triphosphate (ATP) by AckA (Hosmer et al. 2024). The Pta–AckA pathway contributes to cellular energy generation (Hosmer et al. 2024), controls cellular concentrations of acetyl‐CoA and acetyl‐phosphate (Ramos‐Montañez et al. 2010; Post et al. 2017; Schütze et al. 2020; Chang et al. 1999; Christensen et al. 2019; Kuhn et al. 2014; Weinert et al. 2013), and can regulate carbohydrate utilization and carbon flux (Wolfe 2005). This incomplete oxidation of carbohydrate to acetate is known as overflow metabolism in bacteria that can also fully oxidize carbohydrate substrates via respiratory metabolism.

Acetyl‐phosphate and acetyl‐CoA may also contribute to epigenetic regulation through their involvement in enzymatic and nonenzymatic protein acetylation (Ramos‐Montañez et al. 2010; Post et al. 2017; Schütze et al. 2020). Enzymatic protein acetylation requires acetyltransferases that use acetyl‐CoA as a substrate, while nonenzymatic protein acetylation is due to the spontaneous reaction of amino acid side chains with acetyl‐phosphate (Christensen et al. 2019; VanDrisse and Escalante‐Semerena 2019). Protein acetylation most commonly occurs at lysine residues and can modulate protein function, stability, and localization in both bacterial and host cells (Christensen et al. 2019; VanDrisse and Escalante‐Semerena 2019). There is also evidence that acetate can act as an immunomodulatory metabolite, where it has been shown to play a role in the suppression of host cell pro‐inflammatory processes, and in aiding bacterial persistence (Hosmer et al. 2023; López‐López et al. 2020; Hosmer et al. 2022; Vinolo et al. 2011; Xu et al. 2019).

Despite its ability to produce large amounts of acetate as a metabolic end‐product, investigations of the role of acetate and acetate production in *H. influenzae* physiology and virulence are scarce. We recently showed that acetate production appears to contribute to immunomodulatory action during *H. influenzae* infection of 16HBE14 tissue cells (Hosmer et al. 2022). In these experiments, acetate reduced the production of pro‐inflammatory cytokines, such as interleukin‐8 (IL‐8), in the presence of live but not heat‐killed NTHi (Hosmer et al. 2022). A pro‐inflammatory effect of acetate on tissue cells had previously been observed using NTHi culture supernatants, which are comparable to the use of heat‐killed bacteria, from strains able to produce acetate compared with a strain that did not produce acetate (López‐López et al. 2020). Here, only acetate‐containing supernatants stimulated IL‐8 and CXCL1 expression in A549 tissue cells (López‐López et al. 2020). The same study also investigated the role of acetate production in the physiology of the laboratory‐adapted NTHi reference strain, RdKW20, and the chronic obstructive pulmonary disease (COPD)‐isolate strain 411. Inactivation of both *pta* and *ackA* through a polar *ackA* mutation caused an ~ 10‐fold decrease in anaerobic (AN) acetate production (López‐López et al. 2020). The metabolic impairment reduced *H. influenzae* growth by 36% under aerobic conditions and by 50% under AN conditions (López‐López et al. 2020). The mutation mildly attenuated RdKW20 virulence in mice, with colony‐forming units (CFUs) of the polar ackA mutant strain recovered from mouse lungs and bronchoalveolar lavage fluid reduced by 10‐ and 13‐fold, respectively, compared with the wild type.

To better define the roles of *pta* and *ackA* in *H. influenzae* physiology and virulence, here we have generated nonpolar, single‐gene mutations in *ackA* and *pta* using the nontypeable COPD‐isolate strain, *H. influenzae* 2019. The metabolic capabilities, oxidative stress responses, and the roles of these genes in host cell colonization and infection persistence were characterized. Our results demonstrate a clear role for acetate production in maintaining *H. influenzae* overall fitness through regulation of carbon flux, energy generation, and redox balance, as well as in intracellular colonization of host cells, thereby affecting potential reservoirs for persistent infection.

## 2 Materials and Methods

### 2.1 Bacterial Strains and Growth Conditions

*Escherichia coli* strains were cultured using lysogeny broth medium. NTHi strain 2019 (Campagnari et al. 1987) and derivative strains (Table S1) were routinely cultivated using Brain‐Heart Infusion (BHI) broth (BBL) or BHI agar supplemented with hemin (10 μg/mL) and β‐NAD (10 μg/mL) at 37°C for 16 h and 200 rpm shaking for broth cultures. Chemically defined growth medium (CDM) (Coleman et al. 2003) was also used and contained 10 mM glucose, 1 mM sodium pyruvate, 786 µM uracil, 7.4 mM inosine, 24 mM NaHCO_3_, 25 mM HEPES pH 7.4, 10 μg/mL β‐NAD, and 10 μg/mL hemin in RPMI1640 (Sigma‐Aldrich). To test the effects of different carbon sources, glucose was replaced with either 25 mM l‐lactate, 10 mM ribose, and 4 mM uridine in the CDM (Dhouib et al. 2016, 2015). Antibiotics were added where required (final concentrations: *E. coli*/Hi: kanamycin 100/20 μg/mL, spectinomycin 50/20 μg/mL, ampicillin 100/–μg/mL). NTHi growth experiments used microtitre plates (200 μL CDM/well), with incubation at 37°C, 200 rpm using a Clariostar multimode plate reader (BMG LabTech). Gas‐phases used in Clariostar experiments were microaerobic (MA) (2.8% O_2_ and 5% CO_2_) and AN (0.5% O_2_ and 5% CO_2_). Growth rates per hour for NTHi were calculated according to the method described by Kurokawa and Ying (2017).

### 2.2 NTHi Transformation

Transformation of NTHi was performed according to the protocol described in Poje and Redfield (2003). *H. influenzae* were transferred twice on BHI agar plates supplemented with hemin (10 μg/mL) and β‐NAD (10 μg/mL) with antibiotics added as required (37°C, 5% CO_2_, and 16 h) before being resuspended in 20 mL BHI broth in a 50‐mL tube to a starting OD_600nm_ 0.05. The cultures were incubated at 37°C with shaking at 200 rpm until an OD_600nm_ of 0.2–0.4 was reached. The cultures were centrifuged at 375*g* for 10 min at room temperature, and the cell pellets were washed with 20 mL pre‐warmed maintenance intravenous (MIV) solution, then settled by centrifugation. The cell pellets were resuspended in 5 mL pre‐warmed MIV solution and incubated at 37°C for 100 min with shaking at 90 rpm. In all, 10 μL of 1 mg of linearized plasmid was added to 500 μL of competent cells and incubated at 37°C for 30 min in a CO_2_ incubator. BHI broth (2 mL) was added, followed by a 1‐h incubation under the same conditions, then the medium and plasmids were removed by harvesting the bacteria as described above and resuspending the pellets in 1 mL of fresh BHI broth. The cultures (50–200 μL) were plated on selective plates and incubated in a CO_2_ incubator at 37°C for 16–24 h.

MIV solution was prepared freshly by combining 25 mL solution 21 (per L: 4 g l‐aspartate, 0.2 g l‐glutamate, 1 g fumarate, 4.7 g NaCl, 0.87 g K_2_HPO_4_, 0.76 g KH_2_PO_4_, 0.2 mL Tween 80, and pH 7.0) with 0.25 mL solution 22 (4 mg l‐cytosine, 10 mg l‐tyrosine dissolved in 1 mL 1 M HCl at 37°C followed by addition of 9 mL H_2_O and 6 mg l‐citrulline, 20 mg l‐phenylalanine, 30 mg l‐serine, and 20 mg l‐alanine), 0.25 mL 0.1 M CaCl_2_, 0.25 mL 0.05 M MgCl_2_, and 0.25 mL 5% Difco vitamin‐free casamino acids.

### 2.3 Susceptibility Assays

NTHi cell material freshly grown on supplemented BHI (sBHI) plates was resuspended to an OD_600nm_ of 1.0 in sterile 1 × phosphate buffered saline (PBS). The bacterial suspension (900 μL) was then combined with 100 μL of a freshly prepared, 10× stock of the test compound, incubated at room temperature with gentle orbital shaking for 1 h, followed by immediate serial dilution (up to 10^−7^) in BHI and plating on sBHI plates. Assays used 100–500 mM H_2_O_2_, 2–5 mM paraquat, or 50–250 μM HOCl as in Nasreen et al. (2020). Each susceptibility assay was repeated on three independent days; on each day, three biological replicates were used for each strain.

### 2.4 Omnilog Phenotypic Microarray (PM)

Carbon, phosphorus, and nitrogen sources used by NTHi strains were determined using Biolog plates PM01, PM02B, PM03A, and PM04A. Additionally, Biolog plates PM09 and PM10 were used to determine the effects of osmolytes and pH levels on the growth of NTHi strains. NTHi strains were passaged twice on sBHI plates before cell material was resuspended in 2–3 mL of IF‐0a or IF‐10b containing the appropriate PM (12×) additives as set out in Table S2. For plates that do not test carbon source utilization, glucose, pyruvate, tricarballylic acid, and yeast extract were included as carbon sources (Table S2). The turbidity of the inoculum was adjusted to 65% T with uninoculated medium as the blank (100% T) before 100 μL of the inoculated 1 × PM solution was added to each well of the corresponding Biolog PM plate. Plates were incubated in the Omnilog incubator at 37°C, and readings were taken every hour for 48 h. Omnilog data collection used Omnilog software Version 3.0.1.b31, data analysis used Omnilog data analysis software Version 1.7.

### 2.5 Biofilm and Biomass Determination

Biofilm formation and bacterial survival within biofilms were tested as described by Dhouib et al. (2016). *H. influenzae* strains were grown in sBHI to OD_600nm_ 0.2–0.3 under MA conditions at 37°C with shaking (200 rpm) broth, 20 μg/mL kanamycin was added where appropriate. Cultures were diluted to an OD_600nm_ of 0.06 in sBHI, 100 μL/well was added to round‐bottom 96‐well plates (TechnoPlas), leaving the edge wells empty, and incubated statically at 37°C, 5% CO_2_, for 16 h. To prevent evaporation, sterile water was added to the edge wells before covering the plate with a lid. Following incubation, planktonic cells were removed by washing twice with sterile water, and the biofilm was stained with 0.1% crystal violet as in Schembri and Klemm (2001). After 10 min, the crystal violet was removed and the plate was left to dry. Once dried, 30% acetic acid was added to dissolve the crystal violet, and the OD_550nm_ was measured using a microtitre plate reader. Viable cells present in biofilms were determined in replicate plates after overnight incubation and removal of planktonic cells by incubating each well with 200 μL of 0.1 mg/mL proteinase K for 10 min at room temperature to dissolve the biofilm, followed by serial dilution in BHI (up to 10^−7^) and plating on sBHI agar to quantify CFU/mL.

### 2.6 General Molecular and Biochemical Methods

Standard methods were used throughout (Ausubel 1987). Hi2019 genomic DNA was isolated using DNAzol (Thermo Fisher Scientific), and oligonucleotides for polymerase chain reaction (PCR) (Table S3) were supplied by Integrated DNA Technology. GoTaq Green MasterMix (Promega) was used for general PCR, PCR product, and plasmid purification used the GeneJET PCR Purification kit (Thermo Fisher Scientific) and the GeneJET Plasmid Miniprep Kit (Thermo Fisher Scientific). Restriction enzymes and T4 ligase were from Thermo Fisher Scientific and New England Biolabs. Protein concentrations were determined using either the Sigma‐Aldrich BCA‐1 Kit or the Pierce Dilution‐Free Rapid Gold BCA Protein Assay Kit (Thermo Fisher Scientific).

### 2.7 Construction of Hi2019^*ΔackA*^ and Hi2019^*Δpta*^ Mutant Strains

To generate single‐gene knockout mutations in *H. influenzae*, Gibson assembly was used to construct plasmids targeting the *ackA* and *pta* genes. Each knockout plasmid was assembled from four DNA fragments: (i) a plasmid backbone (pBluescript II SK+), (ii) ~ 1 kb upstream of the kanamycin cassette insertion site in the target gene, (iii) ~ 1 kb downstream of the kanamycin cassette insertion site in the target gene, and (iv) a kanamycin resistance cassette from pUC4K. Primers contained 20‐bp overhangs homologous to other fragments, as required for Gibson assembly (Table S3). All fragments were PCR amplified, and DpnI digestion was used to remove template DNA. Plasmids were assembled using the NEBuilder HiFi DNA Assembly Kit (New England Biolabs) according to the manufacturer's instructions with equimolar amounts of each fragment (typically 0.2–0.5 pmol total DNA in a 20‐μL reaction) and transformed into chemically competent *E. coli* NEB5alpha. The resulting constructs, pBlu‐Hi*ackA::*kan and pBlu‐Hi*pta::*kan, were verified by PCR.

Knock‐out plasmids were linearized by restriction digestion and transformed into Hi2019 as described in Poje and Redfield (2003) to generate the Hi2019^Δ*ackA*^ and Hi2019^Δ*pta*^ strains. All mutant strains were verified by PCR to confirm the mutation. To complement the Hi2019^Δ*ackA*^ and Hi2019^Δ*pta*^ strains, the primers p601.1_Hi_AP_GB_F and p601.1_Hi_AP_GB_R were used to amplify a 3375‐bp region covering the *ackA–pta* operon, including a 200‐bp promoter region upstream of *ackA*. The amplified fragment was then cloned into the plasmid p601.1‐Sp2 (Johnston et al. 2007) using the NEBuilder HiFi DNA Assembly Kit to construct p601.1_Hi‐*ackA–pta*. Despite multiple attempts and variations of transformation conditions by, for example, using precultures grown on either sBHI or complete CDM, no complemented Hi2019^*ΔackA*^ or Hi2019^*Δpta*^ strains were obtained. Alterations to the incubation period in competence‐inducing medium (MIV) (Poje and Redfield 2003) and the spectinomycin concentration used for selection were also trialed but did not lead to successful transformations. As the transformation of *H. influenzae* relies on the development of DNA‐uptake competence during incubation in the MIV medium, it was hypothesized that this process might be impaired in Hi2019^Δ*ackA*^ and Hi2019^Δ*pta*^ strains. RNA from Hi2019^WT^, Hi2019^Δ*ackA*^, and Hi2019^Δ*pta*^ strains was isolated following incubation in MIV medium during transformation (Poje and Redfield 2003), complementary DNA (cDNA) generated using Superscript IV VILO Mastermix (Thermo Fisher Scientific), and expression of known competence genes determined using quantitative PCR (qPCR). This revealed that genes, such as *comCEF*, which regulate competence, or genes required for DNA binding during transformation (e.g., *dpr*), exhibited a 2.5–10‐fold reduction in expression in the mutant strains (Figure S1B). We propose that the reduced expression of competence genes is a major reason why complementation could not be achieved, as the mutant strains were unable to develop sufficient DNA‐uptake competence; however, there may be other possible explanations for this phenomenon, including the lack of energy generation or the altered redox balance in the mutant strains.

### 2.8 NAD^+^/NADH and NADP^+^/NADPH Detection

Nicotinamide adenine dinucleotide (NAD^+^), reduced NAD^+^ (NADH), and nicotinamide adenine dinucleotide phosphate (NADP^+^), NADPH levels in NTHi cells were determined using the NAD/NADH‐Glo and NADP/NADPH‐Glo Assay Kits (Promega) following the manufacturer's instructions. For both assays, NTHi strains were grown in complete CDM under MA conditions and harvested at an OD_600nm_ of 0.6. In all, 100 µL of 2 × 10^9^ cells/mL (2 × 10^8^ cells) were lysed according to the manufacturer's instructions, and detection reagent was added. The detection reagent was incubated with the lysates for 45 min at room temperature, and luminescence was measured using a Clariostar plate reader (BMG LabTech). Standard curves ranging from 5 to 400 nM were prepared for all analytes as described by the manufacturer.

### 2.9 ATP Detection

ATP levels were determined using the BacTiter‐Glo Microbial Cell Viability Assay kit (Promega) according to the manufacturer's instructions. Briefly, after MA growth to OD_600nm_ 0.6 in complete CDM, 100 μL of 10^8^ CFU/mL (OD_600nm_ 0.015, 100 μL contains 10^7^ bacterial cells) was combined with 100 μL of BacTiter‐Glo Reagent at room temperature in a 96‐well plate (Greiner Bio‐One). A standard curve was generated using 10‐fold serial dilutions (1–0.0001 µM) of an ATP stock solution (1 mM, New England Biolabs) prepared in CDM. Shaking for 1 min at 100 rpm in a Thermomix C (Eppendorf) was used to ensure all samples were mixed. Luminescence was measured immediately using a Clariostar plate reader (BMG LabTech). ATP concentrations in samples were determined relative to the standard curve.

### 2.10 Enzymatic Determination of Acetic Acid and d‐Lactate

For enzymatic determinations of acetate and d‐lactate, 1 mL of *H. influenzae* cultures grown for 16 h in complete CDM under MA conditions was pelleted (3 min, 4°C, and 22,000*g*); the supernatant was transferred into new tubes and stored at −20°C until use. Acetate and d‐lactate concentrations in these supernatants were determined using acetate and d‐lactate determination kits (Megazyme) according to the manufacturer's instructions. Fresh CDM was used as the negative control.

### 2.11 NTHi Host–Cell Adherence and Invasion Assays Using 16HBE14 Tissue Cells

Human bronchial epithelial 16HBE14 cells (Gruenert et al. 1988) were cultured in Minimal Essential Medium (MEM) with 10% heat‐inactivated fetal bovine serum (FBS) (Thermo Fisher Scientific). NTHi adherence and invasion assays were performed using a multiplicity of infection (MOI) of 100:1 and 4 and 24 h infection times as described in Dhouib et al. (2016). To quantify total adherent bacteria, infected monolayers were washed three times with MEM to remove nonadherent bacteria, followed by lysis with 100 μL 0.1% (w/v) saponin for 10 min at 37°C. For intracellular bacterial counts, 50 μg/mL gentamicin was added to the MEM medium and infected monolayers incubated for 1 h to kill extracellular bacteria, followed by washing and cell lysis. Bacterial counts (CFU/mL) were determined by serial dilution and plating on sBHI.

### 2.12 NTHi Infections of Normal Human Nasal Epithelia (NHNE)

NHNE was prepared as in Yeo et al. (2019) from primary nasal cells donated by a healthy donor, AV082. Primary nasal cells were seeded at an initial density of 39,000 cells/transwell insert and cultured in submerged culture using Pneumacult‐EX medium (Stemcell Technologies) until confluence was reached. Cells were airlifted using Pneumacult‐ALI medium (Stemcell Technologies) in the basal chamber only and cultivated into well‐differentiated epithelia as indicated by the presence of beating cilia under light microscopy. NTHI infections were carried out 30 days post‐airlift. Transepithelial electrical resistance (TEER) measurements were carried out using an EVOM2 epithelial voltohmmeter (World Precision Instruments) equipped with an STX2 electrode. For the measurement, 100 μL of 1 × PBS was added to the apical chamber of fully differentiated NHNE. Final TEER values were calculated after subtracting the background resistance from inserts without cells. Seven days prior to infection, the differentiated NHNE was transferred to steroid‐ and antibiotic‐free PneumaCult‐ALI medium (Stemcell Technologies).

NHNE apical surfaces were washed with 100 μL of 1 × PBS every 2 days, and the basal medium was replaced every 2 days for the duration of the experiment. For infection, NTHi grown on sBHI plates overnight were resuspended in 1 × PBS to an initial OD_600_ of 0.15 and used to infect NHNE on the apical side at MOI 10:1. Uninfected control NHNE was exposed to an equal volume of sterile 1 × PBS. The inoculum was removed after 24 h by washing with 200 µL 1 × PBS, and the basal medium was also replaced. For total bacterial counts, infected NHNE was washed five times with 1 × PBS apically (200 μL) and basally (1 mL), and then incubated with 100 μL of 0.1% (w/v) saponin at 37°C for 10 min. Bacterial loads in lysates were determined by plating serial dilutions of the saponin lysate on sBHI agar. Intraepithelial bacterial CFU were determined after treating the NHNE apically and basally with gentamycin (100 μg/mL) for 1 h at 37°C. Gentamicin was then aspirated, and the infected NHNE was washed five times with 200 μL 1 × PBS in the apical chamber and 1 mL in the basal chamber before being lysed with 100 μL of 0.1% saponin.

For competition assays, NHNE was infected with an equal mixture of Hi2019^WT^ and one mutant strain (MOI 10:1 in total) and bacterial loads analyzed as described above, except that serial dilutions were plated on sBHI and on sBHI with 20 μg/mL kanamycin to determine total bacterial CFU/mL and CFU/mL of NTHi carrying the mutation, respectively. Hi2019^WT^ cell numbers were determined by subtracting mutant strain CFU/mL from total NTHi CFU/mL for the same time point and used to compute fold‐changes. Each infection and coinfection used two biological replicates for each strain or strain combination and time point. For each biological replicate, three technical replicates were used to determine CFU/mL.

### 2.13 Mouse Bone Marrow Macrophage (BMM) Infection Assay

Infection assays using mouse BMMs were performed as in Nasreen et al. (2024). Isolation of BMMs from C57BL/6 mice was adapted from Tushinski et al. (1982). Briefly, mice were sacrificed, and femurs and tibias were collected after removing muscles and surrounding tissues. The bone cavities were flushed with complete macrophage medium (RPMI1640) containing 10% heat‐inactivated FBS, 2 mM l‐glutamine (GlutaMAX, Thermo Scientific), and 150 ng/mL recombinant colony‐stimulating factor‐1 (rCSF‐1, produced at Protein Expression Facility, The University of Queensland). The isolated BMMs were stored in liquid nitrogen (−196°C).

BMMs were washed twice with complete medium (300*g* for 5 min, RT) before being evenly distributed into eight sterile 100 mm square Petri dishes, each containing 15 mL of complete medium with 1% penicillin/streptomycin (Thermo Fisher Scientific). The plates were incubated for 6 days at 37°C with 5% CO_2_. On day 3, 150 ng/mL rCSF‐1 was added to the plates. On day 6, the growth medium was discarded, and BMMs were washed off the plate using 10 mL of 1 × PBS. BMM were then centrifuged at 300*g* for 5 min at room temperature (RT) and resuspended in antibiotic‐free BMM medium. In all, 1 × 10^5^ cells/well were seeded into 24‐well tissue culture plates and incubated at 37°C with 5% CO_2_ for 18 h prior to the infection assay. For infection, bacteria were added at MOI 1000:1. After 1 h, the inoculum was removed, followed by two washes with RPMI and addition of complete macrophage medium with 7 μg/mL polymyxin B to the wells for 60 min to remove extracellular bacteria. For 2 h infections, cell layers were washed three times with RPMI directly after the incubation with polymyxin B, before 100 μL of 0.1% saponin was used to lyse the macrophages. Intracellular bacteria were quantified through dilution plating. For 6 h infections, the medium containing 7 μg/mL polymyxin B was replaced with medium containing 0.5 μg/mL polymyxin B for 4 h before processing the infected BMMs as described to determine intracellular bacterial numbers.

For 30 min attachment/adherence assays, BMMs were incubated with bacteria for 30 min before the inoculum was removed, and the BMMs were washed three times with RPMI. Macrophages were lysed using 100 μL of 0.1% saponin, and bacterial counts were quantified by serial dilution plating.

### 2.14 ELISA Assays

Supernatants from 16HBE14 and NHNE infection assays were used for ELISA (IL‐8, Uncoated ELISA kit, Thermo Fisher Scientific) following the manufacturer's instructions. The supernatants from 16HBE14 and NHNE infections were diluted 50‐ and 100‐fold, respectively.

### 2.15 Western Blot

*H. influenzae* was grown in complete CDM for 16 h under MA or AN conditions, and the bacteria were harvested by centrifugation at 2369*g* for 10 min at 4°C. Supernatants were discarded, and the cell pellets were stored at −20°C until further use. Cell extracts were prepared using B‐PER Bacterial Protein Extraction Reagent (Thermo Fisher Scientific) according to the manufacturer's protocol with inclusion of lysozyme and DNase. Total protein (30 µg) per lane was separated on Bolt Bis–Tris Plus Mini Protein Gels, 4%–12% (Thermo Fisher Scientific), and proteins were transferred to a nitrocellulose membrane using Power Blotter Select transfer stacks and the “mixed MW” transfer program on a Power Blotter system (Thermo Fisher Scientific). Membranes were briefly stained with 1% Ponceau S (w/v) in 5% (v/v) acetic acid to visualize proteins, followed by destaining using 25 mM NaOH. Membranes were blocked using 1 × Blocker FL Fluorescent protein block (Thermo Fisher Scientific) for 60 min at room temperature. The primary antibody, Acetylated Lysine Recombinant Monoclonal antibody (MA5‐33031, Thermo Fisher Scientific) was added to the blocking buffer (dilution: 1:1000), followed by incubation at 4°C for 16 h. Following incubation, the membrane was washed five times with 1 × PBS before incubation with the secondary antibody (Anti‐Rabbit–Alexa conjugate antibody, cat no. A27041, Thermo Fisher Scientific, dilution: 1:10,000 in 1 × Fl Blocker) for 1 h at room temperature. The membranes were washed five times with 1 × PBS and imaged immediately using an Odyssey CLX imaging system (Li‐Cor Biosciences).

### 2.16 RNA Isolation

Samples were collected from Hi2019^WT^, Hi2019^Δ*ackA*^, and Hi2019^Δ*pta*^ during midexponential growth (Hi2019^WT^ OD_600nm_ 0.67, Hi2019^Δ*ackA*^ OD_600nm_ 0.61, and Hi2019^Δ*pta*^ OD_600nm_ 0.6) on complete CDM. RNA from each sample was preserved using RNAprotect Bacteria Reagent (Qiagen) and isolated using the Illustra RNAspin Mini RNA isolation kit (Cytiva). Genomic DNA (gDNA) was removed using the Turbo DNAfree Kit (Thermo Fisher Scientific), and RNA concentrations were determined using a Quant‐it RNA HS kit (Thermo Fisher Scientific). Removal of gDNA was confirmed by PCR using gyrase (*gyrA*) primers (Table S3) with purified RNA as the template. RNA was also isolated from Hi2019^WT^, Hi2019^Δ*ackA*^, and Hi2019^Δ*pta*^ following incubation in MIV medium, which induces competence during the transformation process.

### 2.17 cDNA Synthesis and Quantitative Reverse Transcription PCR (qRT‐PCR) Analyses

cDNA was synthesized from 500 ng of gDNA‐free RNA using either the Superscript IV VILO Mastermix (Thermo Fisher Scientific) or the LunaScript RT SuperMix Kit (New England Biolabs) and RNasin Ribonuclease Inhibitors (Promega). Each qRT‐PCR reaction (10 μL) comprised 2 μL of 1:100 diluted cDNA as template, 5 μL of QuantiNova SYBR Green qPCR master mix (Qiagen), and 2 μL of specific oligonucleotide primers (conc., 3.5 μM, Table S3). Gyrase (*gyrA* gene) was used as the reference gene for *H. influenzae*. qPCRs were run on a QuantStudio Flex 6 (Life Technologies). Data analysis and normalization were performed as in Kappler et al. (2005). The cycle threshold values for all samples were determined using QuantStudio Real‐Time PCR software version 1.3 (Thermo Fisher Scientific). PCR efficiencies were determined using LinRegPCR software version 2016.0 (Ruijter et al. 2009).

### 2.18 NMR Metabolomics

Samples (1 mL) of bacterial culture supernatant were collected after 24 h of growth in AN and MA conditions in complete CDM. Bacterial cells were removed by centrifugation, and the media were transferred to a fresh 1.7‐mL Eppendorf tube. Supernatant samples were preserved using 2% (w/v) sodium azide (final conc., 0.01%) and prepared for ^1^H‐NMR analysis as described in Othman et al. (2014). One‐dimensional Carr–Purcell–Meiboom–Gill proton NMR spectra were acquired as in Daubney et al. (2024) on a Bruker AV900 900 MHz spectrometer. Spectra were measured at 298 K using 256 scans, and a fixed spin–spin relaxation delay 2*nτ* of 20 ms (*τ* = 500 μs) was used to eliminate broad signals from high‐molecular‐weight analytes. One‐dimensional spectra were processed using Topspin 3.2 (Bruker Biospin), and metabolites were identified with Chenomx NMR Suite 8.6 (Chenomx Inc., Edmonton, Canada).

### 2.19 Statistical Analyses

Data are presented as means ± SD. To determine differences between groups, depending on the type of data and comparison required, one‐ or two‐way analyses of variance (ANOVAs) or two‐tailed *t* tests were performed. Statistical analyses were performed using the GraphPad Prism 9 software (GraphPad Software Inc., CA, USA). A *p* < 0.05 was generally considered statistically significant; statistical tests were chosen depending on the structure of the data under analysis.

## 3 Results

### 3.1 Hi2019 Mutants in *ackA* and *pta* Reveal Significant Perturbations of the Metabolic Network

Our previous study of acetate metabolism in *H. influenzae* used a polar mutation in the gene encoding acetate kinase (*ackA*) that removed activities of both *ackA* and phosphate acetyltransferase (*pta*) (López‐López et al. 2020), effectively creating a double mutation. For this study, we created nonpolar single‐gene *ackA* and *pta* mutations in an *H. influenzae* COPD isolate, Hi2019, to elucidate the contribution of each enzyme. Mutant strains, denoted as Hi2019^Δ*ackA*^ and Hi2019^Δ*pta*^, were verified by qPCR, revealing that the *ackA* mutation did not affect expression of the downstream *pta* gene (Figure S1A). Despite multiple attempts, the mutant strains could not be complemented (see methods), which, based on our investigations, could be due to a failure to induce DNA‐uptake competence–related genes during transformation (Figure S1B). To compensate for this limitation, all physiological and infection experiments used at least three biological replicates.

**Figure 1:**
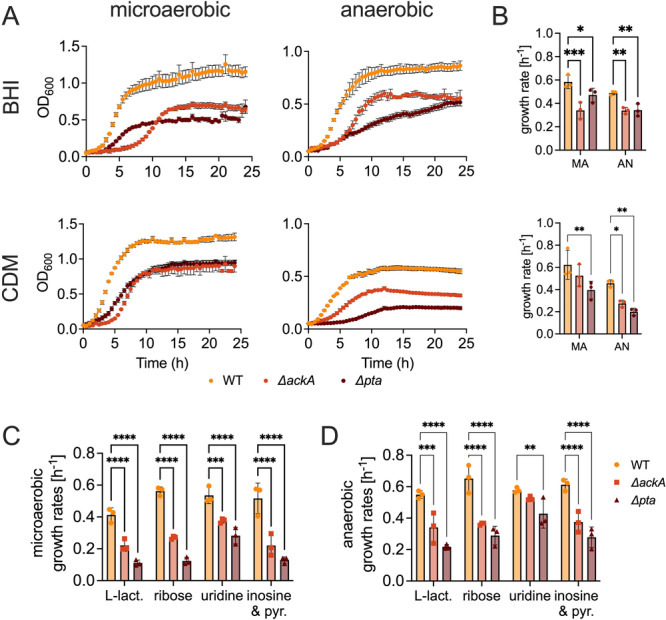
Growth of Hi2019 wild‐type (WT), *ackA*, and *pta* mutant strains under different conditions. (A) Growth on Brain‐Heart Infusion (BHI) and chemically defined medium (CDM) with glucose, inosine, and pyruvate as carbon sources under microaerobic (MA) and anaerobic (AN) conditions. (B) Growth rates on BHI (top) and CDM (bottom). (C) Growth rates on alternative carbon sources under MA conditions. (D) Growth rates on alternative carbon sources under AN conditions. Statistical analyses of growth rates used a two‐way analysis of variance with Dunnett's multiple‐comparison correction. *p* values: *< 0.05; **< 0.01; ***< 0.001; ****< 0.0001.

To assess the impact of the mutations on strain physiology, Hi2019^WT^, Hi2019^Δ*ackA*^, and Hi2019^Δ*pta*^ were initially cultured on the complex medium, sBHI. During MA growth on sBHI, final optical densities of Hi2019^Δ*ackA*^ and Hi2019^Δ*pta*^ were reduced to 54% and 43% of Hi2019^WT^, respectively, and to ~ 73% of wild‐type levels for both strains under AN condition (Figure 1A), while the growth rates of the mutant strains were between 50% and 60% of the wild‐type values (Figure 1B and Table S4). We also observed an extended lag phase for Hi2019^Δ*ackA*^ under MA conditions (Figure 1A).

Growth of the mutant strains was further tested on CDM, which contains glucose as the main carbon source, and on carbon‐source‐modified CDM containing either l‐lactate, ribose, or uridine instead of glucose. In addition to the primary carbon sources, both CDM and carbon‐source‐modified CDM contained uracil, NAD^+^, and hemin, essential nutrients for NTHi, as well as pyruvate and inosine as accessory carbon sources (Field; Coleman et al. 2003; Marshall et al. 2016). Compared with the wild‐type strain, growth on CDM glucose medium was reduced for both Hi2019^Δ*ackA*^ and Hi2019^Δ*pta*^, similar to what we observed in sBHI medium (Figure 1A). Growth rates were reduced to 58% and 47% of wild‐type rates, respectively, for MA conditions, and to 44% and 29% under AN conditions (Figure 1B and Table S4). In keeping with this, final ODs reached only 60%–70% of the wild‐type levels except for Hi2019^Δ*pta*^ where AN growth was only 28% of wild‐type levels. These results are largely similar to those we reported previously for a polar *ackA* mutant in strains RDKW20 and NTHi 411 (López‐López et al. 2020). When CDM was used without a main carbon source, the CDM accessory carbon sources pyruvate and inosine supported growth of Hi2019^WT^, but only minimal growth was observed for the two mutant strains, Hi2019^Δ*ackA*^ and Hi2019^Δ*pta*^ (Figures 1C,D and S2, and Table S4). We then tested the effects of replacing the glucose in CDM with l‐lactate, ribose, or uridine, carbon sources known to support robust growth of NTHi strains (Hosmer et al. 2022). l‐lactate is directly metabolized to pyruvate, a main precursor for many metabolic processes, including energy generation via the formation of acetate. Accordingly, growth of both mutant strains was impaired under both MA and AN conditions, with growth rates reduced to 54% (MA) and 62% (AN) of wild‐type levels for Hi2019^Δ*ackA*^, while for Hi2019^Δ*pta*^, growth rates were reduced to 27% (MA) and 39% (AN) (Figure 1C,D).

Ribose is metabolized via the pentose‐phosphate pathway (PPP), and the nucleoside uridine provides both ribose and the nucleobase uracil for use as carbon sources. The wild‐type strain grew well on ribose, but the growth rates for Hi2019^Δ*ackA*^ were reduced to ~ 50% under MA and AN conditions, while for Hi2019^Δ*pta*^, growth rates were reduced to 22% (MA) and 44% (AN) of wild‐type levels. Interestingly, during growth on uridine, growth of the mutant strains was stronger than for ribose alone, suggesting that in addition to ribose, the uracil present in uridine might also contribute to their growth (Figures 1C,D and S2, and Table S4).

Overall, the Hi2019^Δ*pta*^ strain showed greater growth impairment, and both mutant strains were more strongly impacted under MA conditions. Together, the growth data indicate that both the *ackA* and *pta* mutants have reduced metabolic capacity, with the metabolic network in Hi2019^Δ*pta*^ being more strongly perturbed than in the Hi2019^Δ *ackA*^ strains, and with the effects increasing in the presence of oxygen.

### 3.2 Changes in Metabolic Routing Caused by Mutation of *ackA* or *pta* Decrease ATP Levels, Alter the Redox State of the Pyridine Nucleotide Pools, and Affect Metabolic Substrate and End‐Product Profiles

**Figure 2:**
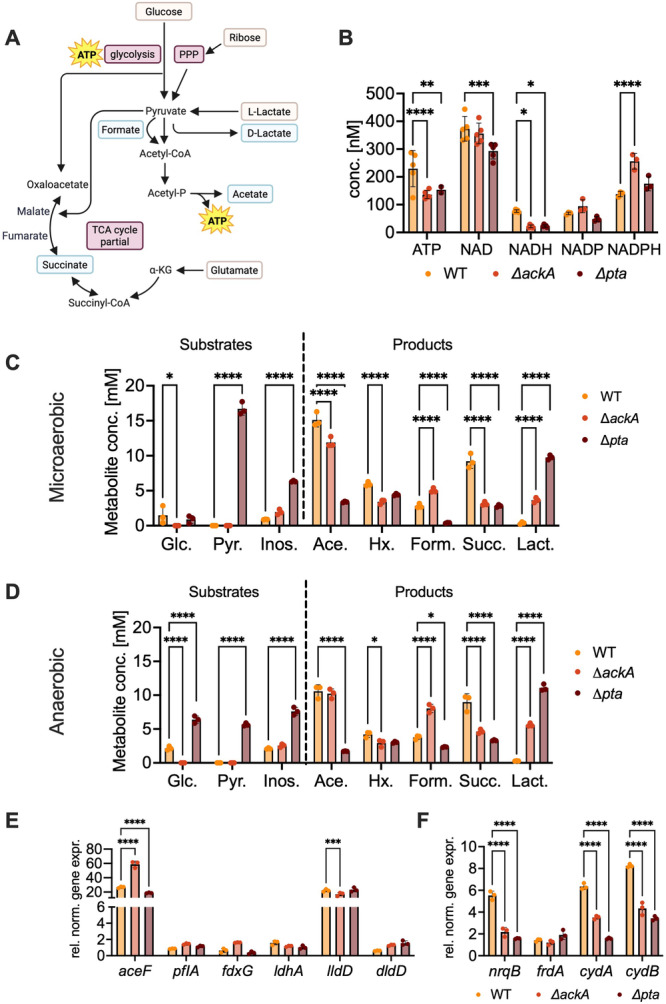
Metabolic properties of Hi2019 wild‐type (WT), *ackA*, and *pta* mutant strains. (A) Schematic representation of *Haemophilus influenzae* central metabolism. (B) ATP, NAD, and NADH concentrations in Hi2019 wild‐type, *ackA*, and *pta* mutant strains growing under microaerobic (MA) conditions. (C, D) Metabolic end‐products produced by Hi2019 wild‐type, *ackA*, and *pta* mutant strains during MA and anaerobic growth. Metabolite concentrations were determined using ^1^H‐NMR. (E, F) qRT‐PCR analysis of genes involved in conversions requiring pyruvate or acetyl‐CoA (E) or the *H. influenzae* respiratory chain (F). Statistical analyses used two‐way ANOVA with Dunnett's multiple‐comparison correction. *p* values: *< 0.05; **< 0.01; ***< 0.001; ****< 0.0001. (A) Created in BioRender. https://BioRender.com/3gcmxhu. ANOVA, analysis of variance; ATP, adenosine triphosphate; NAD, nicotinamide adenine dinucleotide; NADH, reduced NAD; NMR, nuclear magnetic resonance; PCR, polymerase chain reaction; PPP, pentose‐phosphate pathway; qRT‐PCR, quantitative reverse transcription PCR; TCA, tricarboxylic acid cycle.

We hypothesized that the absence of a functional *pta*–*ackA* pathway should reduce ATP production and might also affect the NAD^+^/NADH and NADP^+^/NADPH balance of the mutant strains, given the absence of a functional TCA cycle in *H. influenzae* (Figure 2A). This was found to be the case: following MA growth on CDM glucose, cellular ATP levels were reduced to 46% and 54% of wild‐type levels in Hi2019^Δ*ackA*^ and Hi2019^Δ*pta*^, respectively, matching observations made for similar mutations in *Staphylococcus aureus*, *Bacillus anthracis*, and *E. coli* (Sadykov et al. 2013; Schütze et al. 2020; Marshall et al. 2016; Won et al. 2021).

Additionally, both mutants showed a reduction in NADH availability (Figure 2B). While in the wild‐type strain, the NAD^+^/NADH ratio was ~ 4.4, it was 18.7 for Hi2019^Δ*ackA*^ and 13 for Hi2019^Δ*pta*^ documenting an overoxidation of the NAD^+^/NADH pool (Figure 2B). Interestingly, the availability of NADPH increased in the two mutant strains, with NADP/NADPH ratios decreasing from 0.5 for the wild‐type strain to 0.37 and 0.27 for Hi2019^Δ*ackA*^ and Hi2019^Δ*pta*^, respectively, due to increasing concentrations of NADPH (Figure 2B).

We then analyzed substrate utilization and metabolic end‐products for growth of the wild‐type and mutant strains on CDM glucose using ^1^H‐NMR (Figure 2C,D). With glucose as the main carbon source and in the presence of oxygen, *H. influenzae* wild‐type strains produce acetate as their major end‐product, while at reduced oxygen concentrations, metabolic end‐products diversify to include formate and succinate (López‐López et al. 2020; Hosmer et al. 2022; Muda et al. 2019; Othman et al. 2014). Hypoxanthine also accumulates in the *H. influenzae* growth medium as strains utilize the ribose present in inosine, which is an accessory carbon source in CDM.

After 24 h of MA growth, for Hi2019^WT^, carbon sources in the medium were mostly depleted. No pyruvate, 0.5–2 mM glucose and ~ 0.9 mM inosine remained in the growth medium, while for AN growth, slightly higher concentrations of glucose (~ 2 mM) and inosine (0.9–2.2 mM) remained after growth. Under both MA and AN conditions, the most abundant metabolic end‐products for Hi2019^WT^ were acetate (> 10 mM), succinate (8–9 mM), and formate (2.5–3.9 mM). Additionally, small amounts of lactate (~ 0.3 mM) were also produced, which matches results from previous studies (López‐López et al. 2020; Hosmer et al. 2022; Muda et al. 2019; Othman et al. 2014). Hypoxanthine was present at ~ 6 mM (MA) and ~ 4 mM (AN), in keeping with the amount of inosine consumed under each condition (Figure 2C,D and Table S5).

In contrast, Hi2019^Δ*ackA*^ completely metabolized not only pyruvate but also glucose under both MA and AN conditions, but consumed slightly less inosine (1.9–2.5 mM remaining). This indicates a greater demand for carbon sources that increase ATP production, for example, via increased glycolysis, which is consistent with the loss of the ATP‐producing reaction of AckA (Figure 2C,D).

Unexpectedly, as the final step of the acetate‐producing pathway is inactive in this strain, the Hi2019^Δ*ackA*^ strain produced acetate under both growth conditions, with final concentrations similar to those in wild‐type samples (Figure 2C,D). Relative to the wild‐type strain, Hi2019^Δ*ackA*^ produced half the levels of succinate, twice the amount of formate, and lactate production increased more than 10‐fold (MA, 3.5 mM; AN, 5.5 mM) (Figure 2C,D). A completely different metabolite pattern was detected for the Hi2019^Δ*pta*^ strain. While the Hi2019^Δ*ackA*^ strain produced acetate, likely from nonenzymatic breakdown of accumulating acetyl‐phosphate, the Hi2019^Δ*pta*^ strain produced very little acetate (MA, 3.4 mM; AN, 1.7 mM). Instead, lactate and pyruvate were the main metabolic end‐products (lactate, ~ 10 mM; pyruvate: MA, 16 mM; AN, 5.6 mM). Only about 3 mM succinate was produced (MA, 31%; AN, 37% of wild‐type levels), and formate was mostly detected for AN conditions (AN, ~ 2 mM, 62% of wild‐type levels; MA, 0.4 mM, 14% of wild‐type levels). In keeping with the altered metabolic end‐product spectrum, carbon utilization patterns were also significantly altered, with glucose being almost completely consumed after MA growth, while 5.5 mM glucose remained after AN growth, and pyruvate accumulated under both conditions rather than being depleted (Figure 2C,D). The changes in acetate and lactate production were independently confirmed using enzymatic detection (Figure S3A), which also showed that d‐lactate accumulated in the growth medium, consistent with lactate production via the *H. influenzae* LdhA‐type, NAD^+^‐dependent d‐lactate dehydrogenase and with previous reports (López‐López et al. 2020).

The increased production of d‐lactate as a metabolic end‐product could explain the observed overoxidation of the cellular NAD^+^/NADH pool, as NADH is required for the production of lactate from pyruvate. The changes in the metabolic end‐products in strains carrying mutations in the *pta*–*ackA* pathway confirm a significant perturbation of the Hi2019 metabolic network at the pyruvate node. Changes in the expression of genes associated with pyruvate conversions were limited to pyruvate dehydrogenase (PDH) (*aceF*), where expression increased for the *ackA* mutant strain and was reduced for the *pta* mutant, and a small reduction in *lldD* expression for the *ackA* strain. Interestingly, both strains showed significantly reduced expression of the sodium‐translocating NADH: Quinone Reductase dehydrogenase and the genes encoding the cytochrome bd oxidase (Figure 2E,F), which could also limit growth and redox balancing in the presence of oxygen and suggests activation of the *H. influenzae* ArcAB system, which is known to regulate respiratory chain components (Buettner et al. 2008; Wong et al. 2007). Neither *ackA* nor *pta* is known to be a target of the ArcAB two‐component regulator system, but a possible trigger for ArcAB could be the changes in redox balance in these mutant strains. In fact, our previous work has shown that *ackA* expression appears largely invariant across oxygen concentrations that alter the redox state, indicating constitutive expression of this major ATP‐generating pathway (López‐López et al. 2020; Othman et al. 2014).

### 3.3 Mutations in *ackA* and *pta* Lead to Subtle Adaptations in the Hi2019 Growth Substrates Profiles

**Figure 3:**
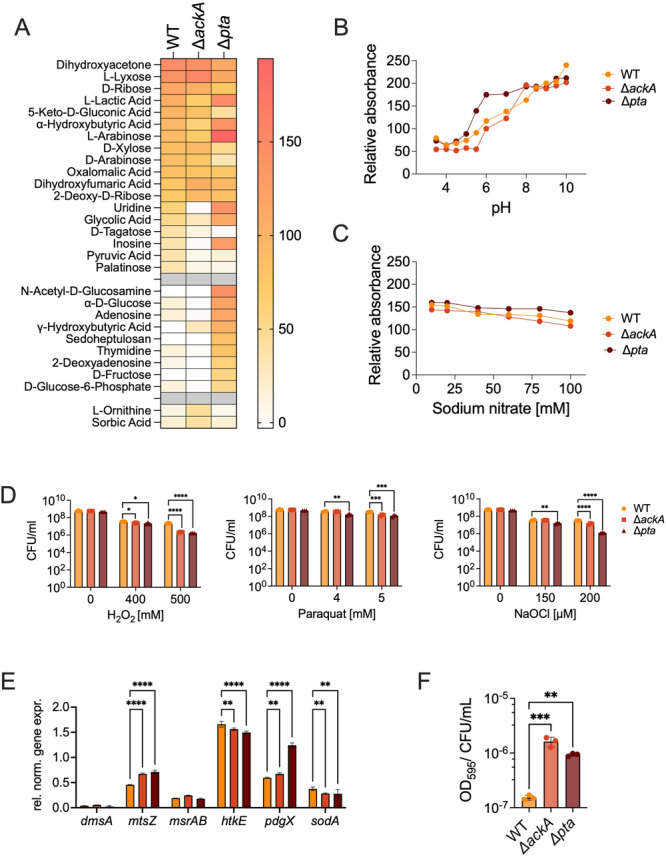
Characterization of growth substrates used by Hi2019 wild‐type (WT), *ackA*, and *pta* mutant strains and their resistance to oxidative stress, including biofilm formation. (A) Heatmap indicating substrate utilization by Hi2019 WT, *ackA*, and *pta* mutant strains. Data are based on Phenotypic Microarray (PM) plates PM1 and PM2. (B) Growth of Hi2019 WT, *ackA*, and *pta* mutant strains at different pH values as determined using PMs. (C) Growth of Hi2019 WT, *ackA*, and *pta* mutant strains in the presence of different concentrations of sodium nitrate as determined using PMs. (D) Resistance of Hi2019 WT, *ackA*, and *pta* mutant strains to different concentrations of hydrogen peroxide, paraquat, and hypochlorous acid. (E) qRT‐PCR analysis of genes involved in oxidative stress resistance in *Haemophilus influenzae*. (F) Normalized biofilm formation for Hi2019 WT, *ackA*, and *pta* mutant strains. Statistical analyses used two‐way ANOVA (D, E) or one‐way ANOVA (F) with Dunnett's multiple‐comparison correction. *p* values: *< 0.05; **< 0.01; ***< 0.001; ****< 0.0001. ANOVA, analysis of variance; qRT‐PCR, quantitative reverse transcription polymerase chain reaction.

Given the strong growth phenotypes observed for substrates such as lactate and ribose, we next used Phenotypic Microarrays (BIOLOG) to further assess the effect of *pta* and *ackA* mutations on nutrient utilization in *H. influenzae*. PMs report bacterial growth in a proprietary medium via a redox dye system that leads to NADH‐dependent color formation and are performed by static incubation of 96‐well plates in a dedicated incubator. Compared with the Hi2019 wild‐type strain that showed strong to moderately strong growth (redox dye readout > 35) with 18 carbon sources, Hi2019^Δ*ackA*^ used 14, and Hi2019^Δ*pta*^ 24 carbon sources (Figure 3A). These carbon sources were only partially complementary and included two and nine carbon sources, respectively, which were more strongly used by the mutant strains. Nucleosides are key nutrients for *H. influenzae* strains; however, for the Hi2019^Δ*ackA*^ strain, we observed reduced growth on nucleosides, while the *pta* mutant showed good growth on various nucleosides and increased growth on glucose (Figure 3A).

There were also some changes in the preferred nitrogen and phosphorus sources: the *ackA* mutant preferred inorganic phosphorus sources, the *pta* mutant strain showed a preference for purine nucleotides, and the wild type was able to use a mix of purine and pyrimidine nucleotides (Figure S4). Nitrogen source utilization followed a similar pattern, where the wild‐type strain utilized mannosamine and several purine and pyrimidine nucleosides, while the *ackA* mutant strains used mannosamine, xanthine, and amino‐caprylic acid, and the *pta* mutant strain had no strongly used nitrogen sources, but instead showed limited use of a broad range of nitrogen‐containing compounds (Figure S4).

The Omnilog‐based analyses reveal extensive changes in the utilization of essential nutrient sources; however, there are some limitations to this assay. The redox dye readout does not directly report growth, which means that substrates that lead to significant redox reactions can cause dye formation even if there is no significant growth. Additionally, the redox dye for use with fastidious bacteria such as *H. influenzae* can already react with several of the substrates in the PM plates, which can either lead to an overestimation of viable growth substrates (e.g., arabinose and deoxyribose), or coincide with actual bacterial growth, as is the case for ribose (Figure S3C). As the *ackA* and *pta* mutant strains show significant perturbation in their redox metabolism, it is possible that some of the nutrient utilization patterns shown here may have been influenced by changes in the level of redox molecule production.

### 3.4 Loss of the *pta*–*ackA* Pathway Does Not Affect Osmotic Stress Resistance but Increases Sensitivity to Oxidative Stress

Changes in flux through metabolic networks, including changes in ATP and NAD^+^/NADH availability, can affect bacterial stress resistance. However, despite the striking metabolic changes caused by abrogation of the *ackA–pta* pathway, tests of sensitivity to pH and osmotic stress in the PM system showed very few changes. For pH‐dependent growth, Hi2019^Δ*pta*^ showed increased redox dye reduction already at pH 5 and reached near maximum dye formation/growth at pH 6, compared with values around pH 8–9 for the wild‐type strains and Hi2019^Δ*ackA*^ (Figure 3B). The two mutant strains also appeared more sensitive to high concentrations of urea, and, in addition, increases in redox dye formation occurred in the presence of sodium formate, NaCl, KCl, and low concentrations of nitrite for Hi2019^Δ*pta*^ (Figures 3C and S5).

In stress‐sensitivity assays, both mutant strains showed statistically significant increases in sensitivity to hydrogen peroxide (500 mM; Δ*ackA*, 10.4‐fold; Δ*pta*, 13‐fold) and paraquat, a superoxide stressor (5 mM; Δ*ackA*, 2.4‐fold; Δ*pta*, 2.7‐fold). The Hi2019^Δ*pta*^ strain was also 25 times more sensitive to 200 μM hypochlorite than the wild type, while Hi2019^Δ*ackA*^ was only 2.4 times more sensitive (Figure 3D).

A possible reason for the increased stress sensitivity in the mutant strains could be underlying changes in the expression of genes involved in oxidative stress defense (Figure 3E). This revealed reduced levels of superoxide dismutase (~ 25% reduction) and catalase (encoded by *hktE*, ~ 10% reduction) expression in both mutant strains, as well as increased expression of peroxiredoxin (*pgdX*, twofold increase) in the *pta* mutant strain. The increase in *pgdX* expression could compensate for the reduction in catalase expression, as both detoxify H_2_O_2_. For the recently described periplasmic stress defense system that includes the sulfoxide reductases MsrAB, MtsZ, and DmsABC (Dhouib et al. 2016; Nasreen et al. 2020, 2024, 2022; Dhouib et al. 2021), expression of all encoding genes was upregulated in the *ackA* mutant strain (28%–48% increase). In contrast, in the *pta* strain, only expression of *mtsZ* was increased (Figure 3E). We propose that the redox perturbations caused by the mutations may, as indicated above, extend to the cellular quinone pool, which would impact the function of the periplasmic MsrAB enzyme, which is required for hypochlorite resistance in *H. influenzae* (Nasreen et al. 2020, 2022). We also tested expression of two key regulators of oxidative stress responses, OxyR and RpoE2, which are activated by hydrogen peroxide and hypochlorite/reactive chlorine species, respectively (Nasreen et al. 2021; Whitby et al. 2012; Harrison et al. 2007) (Figure S6A). Both mutant strains showed reduced *oxyR* expression, which may explain their increased sensitivity to H_2_O_2_. Additionally, expression of the *rpoE2* gene that encodes a main regulator of the periplasmic stress defense system (Nasreen et al. 2021) was increased in Hi2019^*ΔackA*^, matching the increased expression of the associated genes in this strain (Figure S6A). While RpoE2 mediates resistance to HOCl and the increased expression of the *rpoE2* gene in Hi2019^*ΔackA*^ correlates with the resistance of the strain to hypochlorite (Nasreen et al. 2021), *rpoE2* expression levels were similar in the wild type and the Hi2019^*Δpta*^ strain that showed increased sensitivity to hypochlorite, revealing additional complexity in the regulation of hypochlorite sensitivity that requires further investigation (Figures 3D,E and S6A).

In addition to oxidative stress resistance, biofilm formation is another virulence‐relevant trait in *H. influenzae*, and here both Hi2019^*ΔackA*^ and Hi2019^*Δpta*^ showed increased biofilm production when normalized to CFUs in the biofilm. The mutant strains showed, respectively, 9.7‐ and 7.8‐fold increases in biofilm formation relative to the wild type (Figures 3F and S6B,C).

### 3.5 *H. influenzae ackA* and *pta* Mutant Strains Show Reduced Intracellular Survival in Models of Epithelial Cell Infection

On the basis of the data presented above, it was hypothesized that the Hi2019^Δ*ackA*^ and Hi2019^Δ*pta*^ mutants would show a reduced ability to infect tissue cells due to the metabolic imbalance caused by the mutations. This was tested using models of NTHi epithelial infection, such as cultured tissue cells and NHNE.

**Figure 4:**
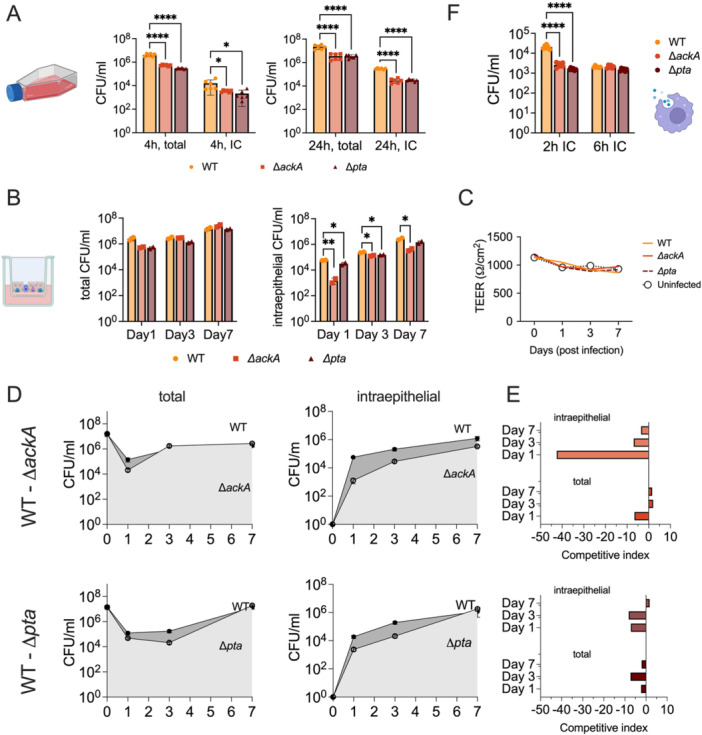
Survival of Hi2019 wild‐type (WT), *ackA*, and *pta* mutant strains in models of epithelial and macrophage infection. (A) Infection of 16HBE14 bronchial epithelial tissue cells for 4 and 24 h. Intracellular colony‐forming unit (CFU)/mL was determined using a Gentamicin protection assay. (B) Seven‐day single *Haemophilus influenzae* strain infections of Normal Human Nasal Epithelia (NHNE) derived from primary human nasal cells. Intraepithelial bacteria were determined using a gentamicin protection assay. (C) Transepithelial electrical resistance (TEER) measurements for NHNE infected with single strains of *H. influenzae*. (D) Competitive Infections (ratio 1:1) of NHNE with the Hi2019 wild type and either the *ackA* or *pta* mutant strains. Intraepithelial bacteria were determined using a gentamicin protection assay. (E) Competitive Indices for competitive infections of NHNE with Hi2019 wild‐type, *ackA*, and *pta* mutant strains shown in (D). (F) Intracellular survival of Hi2019 wild‐type, *ackA*, and *pta* mutant strains in primary, bone‐marrow‐derived murine macrophages at 2 h and 6 h p.i. Extracellular bacteria were removed following a 1‐h infection time using treatment with gentamicin. Statistical analyses used two‐way ANOVA with Dunnett's multiple‐comparison correction. *p* values: *< 0.05; **< 0.01; ***< 0.001; ****< 0.0001. Icons created in BioRender.com.

In keeping with these expectations, infections of submerged bronchial epithelial (16HBE14) cells showed a systematic reduction in total tissue‐associated and intracellular bacterial counts for both Hi2019^Δ*ackA*^ and Hi2019^Δ*pta*^. Compared with wild‐type levels, at 4 h postinfection (p.i.), total tissue cell‐associated bacteria were 7.5‐ and 14‐fold reduced for Hi2019^Δ*ackA*^ and Hi2019^Δ*pta*^, respectively, while intracellular bacteria were reduced 4.6‐ and 7.4‐fold (Figure 4A). By 24 h p.i., the mutant strains showed 6.2‐ and 6.4‐fold reductions in total tissue cell‐associated bacteria, respectively, and increased 9.9‐ and 9.4‐fold reductions in intracellular bacteria (Figures 4A and S7A,B). The continuing decrease in intracellular bacterial populations for the two mutant strains demonstrates that the absence of the *pta*–*ackA* genes reduces bacterial fitness in this niche. Interestingly, for Hi2019^Δ*pta*^, total tissue cell‐associated CFU/mL were more strongly reduced than intracellular bacterial numbers at 4 h p.i. This suggests that this strain may additionally have a defect in tissue cell adherence (Figures 4A and S7A,B), as planktonic cell numbers of Hi2019^Δ*pta*^ at 4 h p.i. were only reduced by ~ 40% compared with the wild type, much less than the ~ 5‐fold reduction observed for the Hi2019^Δ*ackA*^ strain at the same timepoint. The reduced planktonic survival of the two mutant strains may have affected the total number of tissue cell‐associated bacteria at 24 h p.i. reductions in planktonic bacteria (*pta*, 6.4‐fold; *ackA*, 5.97‐fold) were very close to the reduction in total tissue cell‐associated bacteria (Figure S7B).

To determine the relevance of these phenotypes to infections of respiratory epithelia, we then performed infections using NHNE (Figure 4B–E). NHNE are derived from primary human nasal cells that form pseudostratified epithelia during differentiation at the air–liquid interface. The differentiated epithelia include ciliated, basal, and mucous cells (Pezzulo et al. 2011; Ren and Daines 2011). NHNE infections are initiated on the air side of the epithelium, similar to the way in which *H. influenzae* infections occur in humans (Hosmer et al. 2022; Kappler et al. 2024).

During a 7‐day infection with single bacterial strains, on day 1 p.i. intraepithelial colonization of NHNE was reduced 37‐fold in Hi2019^Δ*ackA*^ (*p* = 0.037, one‐way ANOVA), 1.8‐fold on day 3 (*p* = 0.016, one‐way ANOVA) and ~ 6‐fold on day 7 (*p* = 0.023, one‐way ANOVA), while for Hi2019^Δ*pta*^ intraepithelial colonization was reduced between 1.5‐ and 1.8‐fold compared with the wild type throughout (days 1 and 3, *p* = 0.031; day 7, n.s.). Total NHNE‐associated bacteria were reduced ~ 5‐fold for both mutant strains on day 1 p.i., and an ~ 2‐fold reduction remained on day 3 p.i. for Hi2019^Δ*pta*^, while on day 7 p.i. Colonization levels were very similar to the wild‐type strain for both mutant strains (not significant) (Figure 4B). The infection did not damage the NHNE structure as shown by the constantly high TEER that matched the profile of uninfected NHNE (Figure 4C).

Competitive NHNE infections with a 1:1 ratio of wild type and a single mutant strain reproduced the changes seen in single‐strain infections but enhanced the observed phenotypes and revealed differences in the infection dynamics of the two mutant strains (Figure 4D,E). For Hi2019^Δ*ackA*^, total NHNE‐associated bacteria were reduced (C.I. –6.59) on day 1, but cell numbers recovered and the strain even slightly outgrew the wild type by day 3 p.i. (Figure 4D,E). Intraepithelial NHNE colonization of Hi2019^Δ*ackA*^ showed a strong attenuation that was most pronounced on day 1 p.i., (C.I. −42.5) and persisted until day 7, albeit with steadily reducing levels of attenuation. In contrast, Hi2019^Δ*pta*^ showed the strongest attenuation in total and intraepithelial NHNE‐associated bacteria on day 3 p.i. However, attenuation decreased over time and by day 7 p.i., total NHNE‐associated and NHNE intraepithelial bacteria were very similar to the wild type, indicating that the fitness of Hi2019^Δ*pta*^ in the intracellular environment increased over time (Figure 4D,E).

On the basis of the data above, it appears that mutations in *ackA* and *pta* affected general and intraepithelial survival of *H. influenzae* in contact with NHNE, particularly in the first 24–72 h of infection. The *ackA* mutant strain was more profoundly affected at 24 h p.i., with total CFU/mL recovering quickly while intracellular colonization remained impaired. In contrast, total and intraepithelial colonization for the *pta* mutant strain followed similar patterns of variation, with the strongest attenuation observed at 72 h p.i. Overall, it appears that contact with epithelial cells alleviated some of the growth limitations observed in the in vitro culture of these two mutant strains. The mechanism for this is unclear at present, but it is likely due to access to an extended range of growth substrates when in contact with epithelial cells.

### 3.6 Loss of *ackA* Reduces Initial Attachment to Macrophages Compared With the Wild‐Type and *pta* Mutant Strains

Given the reduced intracellular survival of both *pta* and *ackA* mutant strains in two different models of epithelial cell infection, we also tested intracellular survival of the Hi2019^WT^ and the two mutant strains in primary bone marrow‐derived murine macrophages isolated from C57BL/6 mice (Figures 4F and S7D). Macrophages were infected with an MOI of 1:1000 for 1 h, after which extracellular bacteria were removed by gentamicin treatment. At 2 h p.i., intracellular bacteria were reduced by 7.6‐fold (*p* < 0.001) for Hi2019^Δ*ackA*^ and 13‐fold (*p* < 0.001) for Hi2019^Δ*pta*^ compared with the wild‐type strain; however, by 6 h p.i., cell numbers for all strains were similar (Figure 4F). The biggest change between 2 and 6 h p.i. was a 10‐fold reduction in the number of wild‐type cells to levels similar to those of Hi2019^Δ*ackA*^, while Hi2019^Δ*pta*^ cell numbers remained at around 60% of wild‐type and Hi2019^Δ*ackA*^ levels at 6 h p.i. (Figure 4).

To determine whether the difference in intracellular bacteria between the wild‐type and mutant strains at 2 h p.i. could be due to reduced bacterial uptake, we performed a 30 min cell attachment test. This revealed a 13.5‐fold reduced cellular attachment for the *ackA* mutant strain, while no statistically significant changes were observed for the *pta* mutant strain (Figure S7D). This indicates a multifactorial mechanism for reduced intracellular survival, including reduced growth efficiency due to strain‐specific metabolic changes and increased susceptibility to oxidative stress, which together enhance macrophage killing. In the *ackA* mutant, reduced attachment to macrophages would have further attenuated intracellular survival. While attachment defects could not be determined in the NHNE infection model, the low intracellular colonization observed with the *ackA* mutant strain on day 1 p.i. might be related to the observed cell attachment defects. However, the data are not directly comparable because the infection models used different sampling time points (2 h vs. 24 h). It is likely that a similar combination of factors that appear to play distinct roles in the physiology of the two mutant strains also governs the differences in epithelial infection dynamics of the *ackA* and *pta* mutant strains.

## 4 Discussion

**Figure 5:**
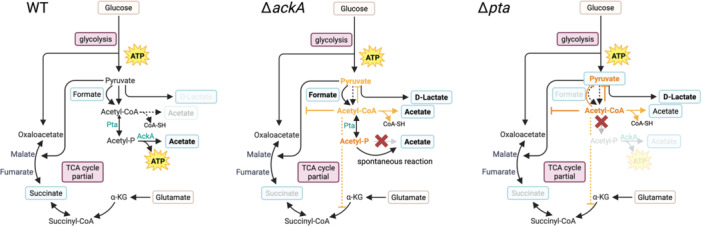
Schematic representation of the metabolic flux changes resulting from the Δ*ackA* and Δ*pta* mutations in *Haemophilus influenzae* during growth on glucose‐containing CDM. *Yellow boxes*, substrates; *purple boxes*, pathway names; *blue boxes*, metabolic end‐products; *yellow or orange metabolite names*, accumulating metabolites; *colored arrows*, proposed reactions; *black arrows*, reactions with confirmed enzymes present in *H. influenzae*; *partially transparent arrows or labels*, inactive reactions or reactions with very low flux. Created in BioRender. https://BioRender.com/3gcmxhu. ATP, adenosine triphosphate; CDM, chemically defined medium; TCA, tricarboxylic acid cycle; WT, wild type.

In *H. influenzae*, acetate is a major metabolic end‐product with known immunomodulatory functions (Hosmer et al. 2022; Othman et al. 2014), but despite this, studies of Pta and AckA, and their roles in *H. influenzae* survival are lacking. This is in contrast to several other human pathogens, where the role of Pta and AckA in bacterial fitness has been extensively studied (Ramos‐Montañez et al. 2010; Sadykov et al. 2013; Post et al. 2017; Schütze et al. 2020; Marshall et al. 2016; Ahn et al. 2020; J. N. Kim et al. 2015), and significant changes in metabolic routing as a result of mutations in *ackA* and/or *pta* have been documented, for example, for *E. coli*, *B. anthracis*, and *S. aureus*. However, a key difference between *H. influenzae* and these bacteria is the absence of a complete TCA cycle in *H. influenzae*, rendering this bacterium incapable of classic overflow metabolism, the context in which mutations in the *pta*–*ackA* pathway have been mostly studied (Sadykov et al. 2013; Schütze et al. 2020; Marshall et al. 2016; Won et al. 2021). Here, we have created nonpolar mutations in both *ackA* and *pta*, revealing that the loss of either enzyme caused significant, but distinct perturbations in *H. influenzae* central carbon metabolism at the central pyruvate and acetyl‐CoA nodes. In *H. influenzae*, *ackA* and *pta* mutations prevented utilization of pyruvate and l‐lactate as carbon sources, as, in the absence of a functional TCA cycle (Figures 2A and 5), these substrates require AckA for ATP production, while substrates such as glucose or ribose that are metabolized via glycolysis or the PPP support growth as they allow substrate‐level phosphorylation through the reaction of pyruvate kinase.

**Figure 6:**
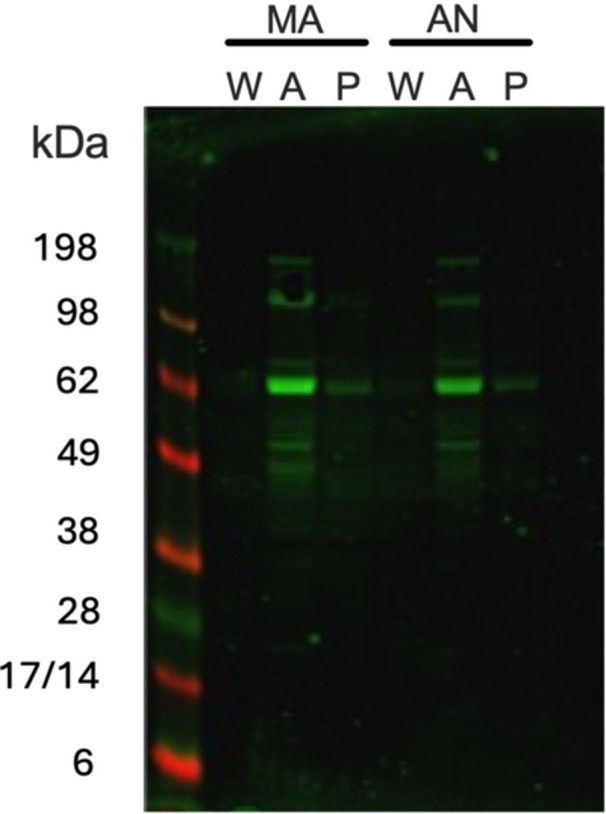
Lysine acetylation in cell extracts derived from Hi2019 wild‐type or the *ackA* or *pta* mutant strains grown under microaerobic (MA) or anaerobic (AN) conditions visualized using a Western blot. W, wild type; A, Δ*ackA* strain; P, Δ*pta* strain.

Analysis of metabolic end‐products for the *H. influenzae ackA* and *pta* mutant strains revealed that modifications of the acetate‐producing pathway did not always abrogate acetate production but instead caused accumulation of pyruvate, which explains other observations, including changes in the NAD/NADH pools. While the Hi2019^Δ*pta*^ strain produced little acetate, matching a polar *ackA* mutant we reported earlier (López‐López et al. 2020), Hi2019^Δ*ackA*^ produced wild‐type levels of acetate. This differs from bacteria, such as *E. coli* and *S. aureus*, where acetate production was reduced in both *ackA* and *pta* mutant strains (Schütze et al. 2020; Marshall et al. 2016), likely because these species have complete TCA cycles that prevent accumulation of Acetyl‐CoA. In *H. influenzae* Hi2019, there are limited options for acetate production in the absence of *ackA*, as the strain lacks an acylphosphatase, and phosphorylation of response regulators by acetyl‐P is well documented, but kinetically slow (*K*_M_ ~ 7–8 mM) (McCleary 1996; Klein et al. 2007). In vitro, the half‐life of acetyl‐P at pH 7 and 37°C is ~ 3 h, suggesting that it is relatively stable in the absence of enzymatic catalysts. However, we propose that the nonenzymatic breakdown still plays a significant role in acetate formation observed in the Hi2019 *ackA* strain, where samples for analysis were collected after 24 h of growth. Additionally, acetate could be formed by a reversal of the Pta reaction that converts acetyl‐P back to Acetyl‐CoA in combination with the reaction of the YciA thioesterase to produce acetate and CoA‐SH. YciA from *H. influenzae* strain Rd has been characterized and is able to convert acetyl‐CoA (Zhuang et al. 2008). An indirect measure of acetyl‐P accumulation in *ackA* gene knockout mutants is an increase in lysine acetylation, which can modulate DNA‐binding, enzyme activity, protein stability, and localization (Christensen et al. 2019; VanDrisse and Escalante‐Semerena 2019). In keeping with results from *ackA–pta* pathway mutants in *E. coli*, *Neisseria gonorrhoeae*, *Klebsiella pneumoniae*, and other bacteria (Wolfe 2005; Post et al. 2017; Schütze et al. 2020; Kuhn et al. 2014; Weinert et al. 2013; Lin et al. 2025), we detected strongly increased protein acetylation in Hi2019^Δ*ackA*^ under both AN and MA conditions, while lysine‐acetylation levels in Hi2019^Δ*pta*^ were only somewhat enhanced, confirming the presence of increased levels of acetyl‐donor molecules in Hi2019^Δ*ackA*^ (Figure 6).

The *H. influenzae ackA* and *pta* mutant strains also produced increased amounts of d‐lactate, and Hi2019^Δ*pta*^ even produced pyruvate as an end product (Figures 5 and S8). Neither of these compounds is a significant product of wild‐type *H. influenzae* metabolism (Hosmer et al. 2022; Muda et al. 2019; Othman et al. 2014), but in the mutant strains is likely driven by the accumulation of acetyl‐CoA (*pta* mutant strain) and acetyl‐P and acetyl‐CoA (*ackA* mutant strain). Increased concentrations of acetyl‐CoA can inhibit PDH, which would then cause accumulation of pyruvate (Schwartz and Reed 1970). In the absence of a functional TCA cycle, accumulated acetyl‐CoA has limited cellular uses, and we found no evidence of increased fatty acid biosynthesis. Lactate production in bacteria normally occurs to alleviate problems with cellular redox balance, such as an overreduction of the NAD^+^/NADH pool. In the *H. influenzae ackA* and *pta* mutant strains, lactate production would be driven by a need to reduce cellular pyruvate concentrations, not an overreduction of the NAD^+^/NADH pool, and therefore increase the oxidation state of the cellular NAD^+^/NADH pool, which matches our observation of increased NAD^+^/NADH ratios in both mutant strains. High NAD^+^/NADH ratios were also documented for *ackA* and *pta* mutants in *S. aureus* and *B. anthracis* (Sadykov et al. 2013; Won et al. 2021). Interestingly, while the NAD^+^/NADH pool was overoxidized in the *H. influenzae ackA* and *pta* mutant strains, the NADP^+^/NADPH pool was overreduced, suggesting either increased production of NADPH, for example, via increased flux through the PPP, or some impairment in NADPH consuming reactions such as fatty acid biosynthesis, oxidative stress responses that require reduction of thioredoxins and glutathione and, to a lesser extent, amino acid biosynthesis. To the best of our knowledge, NADP^+^/NADPH concentrations have not previously been reported for bacterial *ackA* or *pta* mutant strains, and their measurement reveals additional complexities in the metabolic perturbations associated with the loss of these reactions.

The accumulation of pyruvate that creates significant redox and carbon flux issues is unique to *H. influenzae pta*–*ackA* pathway mutant strains, and differs both from results obtained with other bacteria that have a complete TCA cycle, and from findings in *Streptococcus pneumoniae*. This respiratory pathogen lacks a TCA cycle and normally produces l‐lactate as its main metabolic end‐product, but can switch to mixed acid fermentation when growing on fermentable sugars such as galactose or when entering the stationary phase (Al‐Bayati et al. 2017; Gaspar et al. 2014). In *S. pneumoniae*, *pta* mutant strains showed no phenotype, as acetyl‐P can also be produced by the reaction of the H_2_O_2_‐producing pyruvate oxidase (*spxB*), which is absent in *H. influenzae* (Ramos‐Montañez et al. 2010). In contrast, *S. pneumoniae* strains with mutations in *ackA* were found to be unstable and quickly acquired suppressor mutations that often mapped to *spxB* and its regulator, thereby reducing the production of endogenous H_2_O_2_ (Ramos‐Montañez et al. 2010).

Interestingly, despite severe metabolic perturbations during in vitro growth and increased oxidative stress susceptibility, both Hi2019^Δ*pta*^ and Hi2019^Δ*ackA*^ showed only moderate attenuation during infection of cultured tissue cells, primary human epithelia (NHNE), and macrophages. We propose that host cell metabolic activities could alleviate some of the metabolic imbalances in the *H. influenzae* mutant strains by removing accumulating metabolic end‐products, such as pyruvate in the *pta* mutant strain, or by providing access to metabolites that are absent from the in vitro growth medium.

In both models of epithelial infection and in primary murine macrophages, intracellular survival of the mutant strains was reduced at early infection timepoints, with intracellular bacterial loads approaching wild‐type levels by day 7 p.i. in the NHNE model. The growth defect in the intracellular niche suggests that the mutant strains were producing insufficient ATP to thrive, which could result from their inability to utilize l‐lactate, which is important during early colonization of host cells (Hosmer et al. 2022), and low bioavailability of substrates such as glucose that allow ATP production by substrate‐level phosphorylation in the absence of the *ackA–pta* pathway. There is only limited data on intracellular survival of *ackA*/*pta* mutants in other bacteria, with a *Salmonella typhimurium pta* mutant showing no defect in intracellular survival in macrophages (Y. R. Kim et al. 2006). Additionally, *pta* was identified as an essential gene in the obligate intracellular pathogen, *Mycoplasma bovis*, that completely lacks a TCA cycle, during a transposon library screen (Premachandre et al. 2024).

The infection experiments also revealed differences in host–pathogen interactions in the two *H. influenzae* mutant strains. In murine macrophages, the *ackA* mutant strain exhibited an adherence defect, which would have contributed to reduced intracellular colonization, while in competitive infections in NHNE, maximum attenuation occurred at different timepoints, namely, 24 h p.i. for the Δ*ackA* strain and 72 h p.i. for the Δ*pta* strain. The mechanism for this is unclear at present, but could be due to the different effects of the mutations on the metabolic network, and we can also not exclude that the adherence defect observed for the *ackA* strain in macrophage infections extends to epithelial cells. The recovery of both mutant strains to wild‐type levels at later infection timepoints indicates that long‐term survival of *H. influenzae* in the host may not require the *ackA–pta* pathway, potentially because bacterial populations have reached an equilibrium and there is only limited growth, reducing ATP demands and thereby making the mutant strains more competitive.

In other bacterial pathogens, mutations in *ackA* and *pta* reduced the ability of *K. pneumoniae* to infect *Galleria mellonella* larvae, and in *Salmonella* reduced the ability to infect mice and chickens (Lin et al. 2025; Y. R. Kim et al. 2006; Barrow et al. 2015). In *Mycobacterium tuberculosis*, accumulation of acetyl‐P in an *ackA* mutant strain reduced virulence by increasing acetylation of the cAMP receptor protein (CRP), which alters CRP function (Di et al. 2023).

There is thus a wide variety of phenotypes, both metabolic and during infection, associated with mutations of the *ackA–pta* pathway in bacteria. We propose that this may be related to fundamental differences in the metabolic pathways present in each bacterium, and particularly the presence or absence of a functional TCA cycle, and the presence of additional reactions that consume acetyl‐CoA, as in AN *E. coli* cultures, where ethanol formation can alleviate effects of acetyl‐CoA accumulation, or *S. pneumoniae*, where the reaction of SpxB can mitigate effects of a *pta* gene knockout by producing acetyl‐P for ATP production (Ramos‐Montañez et al. 2010; Schütze et al. 2020). Given this diversity, the usefulness of AckA and Pta as antimicrobial drug targets that has been impressively demonstrated in a recent drug screen for new *M. tuberculosis* inhibitors (Subramaniyan et al. 2023) will depend on the metabolic make‐up of the target organism, and may well be species‐ or genus‐specific, avoiding large‐scale effects on the entire human microbiota that are common with traditional antibiotics. For *H. influenzae*, the development of specific *ackA* or *pta* inhibitors could prevent colonization, especially when used alongside conventional antimicrobial therapy during the early stages of infection.

Our work contributes fundamental new insights into the *H. influenzae* metabolic network and the importance of the pyruvate node for optimal functioning of central carbon metabolism, where the oxidative branch of the TCA cycle is absent, a property that is common not only in bacterial pathogens related to *H. influenzae* but also in a range of Gram‐positive bacteria.

## Author Contributions

**Marufa Nasreen:** investigation, writing – original draft, writing – review and editing, formal analysis, visualization, methodology. **Jennifer Hosmer:** investigation, writing – original draft, visualization, writing – review and editing, formal analysis, methodology. **Saurab Kishore Munshi:** investigation, writing – review and editing. **Riya Joshi:** formal analysis, writing – review and editing, visualization. **Ayaho Yamamoto:** methodology, investigation, writing – review and editing, supervision. **Horst J. Schirra:** methodology, formal analysis, writing – review and editing, investigation, supervision. **Peter Sly:** supervision, methodology, writing – review and editing, data curation. **Alastair G. McEwan:** funding acquisition, conceptualization, writing – review and editing, visualization, supervision, data curation, methodology. **Ulrike Kappler:** funding acquisition, conceptualization, formal analysis, visualization, writing – original draft, writing – review and editing, project administration, supervision, data curation, validation, methodology.

## Ethics Statement

Human nasal cells were sampled and donated by healthy donors at the Child Health Research Centre, the University of Queensland, Brisbane (Ethics approval number HREC/20/QCHQ/61894). Experimental animal procedures were conducted in strict accordance with the recommendations outlined in the QLD Animal Care and Protection Act (2001) and the Australian Code of Practice for the Care and Use of Animals for Scientific Purposes, 8th edition. The protocols were approved by the Animal Care and Ethics Committees of QIMR Berghofer and the University of Queensland (Ethics approval number QIMR P3982; ratified as UQ 2019/AE000050).

## Conflicts of Interest

None declared.

## Supporting information

Supporting File 1

Supporting File 2

## Data Availability

All data associated with the manuscript are either presented in the manuscript or as part of the associated supplementary files.
