# Supplementary material for: Acetate‐Linked Energy Metabolism as a Determinant of Early Haemophilus influenzae Infection Fitness
Source: Microbiologyopen. 2026 Jun 14;15(3):e70324. doi: 10.1002/mbo3.70324 (PMC13265617; doi:10.1002/mbo3.70324)
Supplement: Supplementary file 1 — Supporting File 1 [file MBO3-15-e70324-s001.docx]

Acetate‑linked energy metabolism as a determinant of early *Haemophilus* influenzae infection fitness

**Supplementary Figures and Tables**

Marufa Nasreen^1*^, Jennifer Hosmer^1*^, Saurab Munshi^1^, Riya Joshi^1^, Ayaho Yamamoto^2^, Horst Schirra^3,4,5^, Peter Sly^2^, Alastair G. McEwan^1^, Ulrike Kappler^1#^

^1^School of Chemistry and Molecular Biosciences, The University of Queensland, St. Lucia, Qld 4072, Australia

^2^Child Health Research Centre, The University of Queensland, South Brisbane, Qld 4101, Australia

^3^School of Environment and Science, Griffith Institute for Drug Discovery, Griffith University, Nathan, Queensland, Australia

^4^Centre for Advanced Imaging, The University of Queensland, Brisbane, Queensland, Australia

^5^Institute for Biomedicine and Glycomics, Griffith University, Southport, Queensland, Australia

**Table S1** Bacterial strains and plasmids used in this study

| **Strain** | **Description** | **Reference** |
| --- | --- | --- |
| *Escherichia coli*  DH5α | F– φ80lacZΔM15 Δ(lacZYA- argF)U169 recA1 endA1 hsdR17(rK–, mK+) phoA supE44 λ– thi-1 gyrA96 relA1, cloning strain | Life Technologies |
| *Escherichia coli*  NEB 5-alpha | *fhuA2::IS2 Δ(mmuP-mhpD)169 ΔphoA8 glnX44 ϕ80d[ΔlacZ58(M15)] rfbD1 gyrA96 luxS11 recA1 endA1 rphWT thiE1 hsdR17* | New England Biolabs |
| *Haemophilus influenzae* 2019 (Hi2019^WT^) | Clinical isolate from a chronic obstructive pulmonary disease patient. Sequence type 321 | [1] |
| Hi2019^∆^*^ackA^* | Hi2019 WT with *ackA* gene disrupted by the insertion of a kanamycin antibiotic resistance cassette (*ackA::kan*) | This study |
| Hi2019*^∆pta^* | Hi2019 WT with *pta* gene disrupted by the insertion of a kanamycin antibiotic resistance cassette (*pta::kan*) | This study |
| **Plasmid** | **Description** | **Reference** |
| pUC4K | Cloning vector used to isolate the kanamycin resistance cassette. Kan^r^, Amp^r^ | [2] |
| pBluescript II SK+ | Cloning vector | Stratagene |
| pBlue-Hi- *ackA::kan* | pBluescript derivative containing the Hi2019 *ackA* gene disrupted by a kanamycin resistance cassette | This study |
| pBlue-Hi-*pta::kan* | pBluescript derivative containing the Hi2019 *pta* gene disrupted by a kanamycin resistance cassette | This study |
| p601.1-sp2 | Cloning vector containing Hi2019 601.1 pseudogene fragments flanking the insertion region and a spectinomycin resistance cassette | [3] |
| p601.1-Hi-*ackA-pta* | p601.1-sp2 derivative containing the Hi *ackA,* *pta*, and promoter gene region | This study |

**Table S2** Media preparation for Phenotypic Microarray studies

| **Composition and Preparation of 12x PM Additive Solutions** | | | | | | | |
| --- | --- | --- | --- | --- | --- | --- | --- |
| **Ingredients** | **Stock Conc.** | **Conc. in PM Additive (12x)** | **Vol. of solutions required for each plate type** | | | | |
|  |  |  | **PM1,2** | **PM3,6,7,8** | **PM4** | **PM5** | **PM9+** |
| Tricarballylic acid (pH7.1) | 800mM | 240mM | - | 6ml | 6ml | 6ml | - |
| MgCl_2_ x 6H_2_O  CaCl_2_ x 2H_2_O | 240mM  120mM | 24mM  12mM | 2ml | 2ml | 2ml | 2ml | 2ml |
| L-arginine, HCl  L-glutamic acid | 3mM  6mM | 300µM  600µM | 2ml | - | 2ml | - | - |
| β-NAD, Na  hypoxanthine  uracil  L-cystine (pH8.5) | 0.2mM  1mM  1mM  1mM | 60µM  300µM  300µM  300µM | 6ml | 6ml | - | - | - |
| Yeast extract | 0.6% | 0.06% | 2ml | 2ml | 2ml | - | 2ml |
| Tween 80 | 0.6% | 0.06% | 2ml | 2ml | 2ml | - | 2ml |
| D-glucose  Pyruvate, Na | 300mM  600mM | 30mM  60mM | - | 2ml | 2ml | 2ml | 2ml |
| Sterile water | N/A | 0 – 60ml | 6ml | - | 4ml | 10ml | 12ml |
| Total volume |  |  | 20ml | 20ml | 20ml | 20ml | 20ml |
| **Recipe for 1x PM Inoculating Fluids from Stock Solutions** | | | | | | | |
| **PM Stock Solution** | | | **PM1,2** | **PM3,6,7,8** | **PM4** | **PM5** | **PM9+** |
| IF-0a GN/GP (1.2x) | | | 20.0ml | 40ml | 10ml | 10ml | - |
| IF-10b GN/GP (1.2x) | | | - | - | - | - | 110ml |
| Redox Dye G (100x) | | | 0.24ml | 0.48ml | 0.12ml | 0.12ml | 1.32ml |
| PM additive (12x) | | | 2.0ml | 4.0ml | 1.0ml | 1.0ml | 11.0ml |
| After preparation of the 1xPM inoculating fluid, Hi cells were resuspended in 5 ml of 1xPM Inoculating Fluid medium, and the inoculum turbidity was adjusted to 65%T using uninoculated medium as the 100%T blank before loading into the assay plate. | | | | | | | |

**Table S3** –Oligonucleotide primers used in this study.

| **Primer Name** | **Sequence (5’ – 3’)** | **Reference** |
| --- | --- | --- |
| HiackA upGBF | GCAGGAATTCGATATCAAGCGATCCAGCGACAGGGGAAGAAAA | This study |
| HiackA upGBR | GAGACACAACGTGGCTTTCCCTTCACGAGAAATGAAGTAGTGAC | This study |
| HiackA dnGBF | CTTCACGAGGCAGACCTCAGGGCAAACCCGCAGACCAAGTC | This study |
| HiackA dnGBR | GAGGTCGACGGTATCGATAATTACGCCATCTTTACCAAAACGTGC | This study |
| HiackA int F | GGCAAACCCGCAGACCAAGTCGGCAAACCCGCAGACCAAGTC | This study |
| HiackA int R | CTTCACGAGAAATGAAGTAGTGAC | This study |
| HiackA GBcompF | GGGTCAAGGGCGAATTATCACTTCAAGCCAATAAAGCCCTTGAAAAG | This study |
| HiackA GBcompR | CATCGCAGTGATAAATAGCGTAAAGAG TACATCAAGC | This study |
| pUC4K_GB_F | CTGAGGTCTGCCTCGTGAAG | This study |
| pUC4K_GB_R | GGAAAGCCACGTTGTGTCTC | This study |
| pBlue_GB_F | GCTTGATATCGAATTCCTGC | This study |
| pBlue_GB_R | TTATCGATACCGTCGACCTC | This study |
| p601.1 GB F v3 | GGCATATGGTATACCTTAAGGT | This study |
| p601.1 GB R v3 | CGGGGTCAAGGGCGAATTAT | This study |
| **qPCR Primers** | | |
| **Primer name** | **Sequence (5’ – 3’)** | **Reference** |
| Hi_QP_fabAF | GAAACAGGTGCTTTCGGTAAAGGATA | This study |
| Hi_QP_fabAR | ACTTCTCCAACGCCTAATGCTCGC | This study |
| Hi_QP_fabBF | TGTTGCCGGAACAATCAAATTAAAC | This study |
| Hi_QP_fabBR | CTAATTGATTATGTGCAGACCCCG | This study |
| Hi_QP_fabGF | TGCGGCAAAAGCGGGTGTGGTTG | This study |
| Hi_QP_fabGR | CGGCTTTAGCTATGTCTTTTGCT | This study |
| Hi_QP_fabHF | CAGGCAAAGTGAAAAAAGCCTTAGT | This study |
| HI_QP_fabHR | TGGCTGAGCTAAAACAAGGGCAT | This study |
| HI_QP0_zwf_F | GCCAGAACCATCATAATAGCC | [4] |
| HI_QP0_zwf_R | GAAACCGTTCAAAACTTGCTC | [4] |
| HI_QP0_aceF_F | TTATGTGAAGACCGCAGTTAAAG | [4] |
| HI_QP0_aceF_R | AATTTACTGAAATCAACTTTTGG | [4] |
| HI_QP0_cydA_F | TTTTAATAATAAACCGAAACCAAGA | [4] |
| HI_QP0_cydA_R | ATCCGTGCTTATGAATTATTTACTCA | [4] |
| HI_QP0_cydB_F | CAAGCATTGATAAGCTAATAA | [4] |
| HI_QP0_cydB_R | GCGTTCCATTCCATTTTAACG | [4] |
| HI_QP0_fdxG_F | AATCAAATGGAACGGTAAAAACT | This study |
| HI_QP0_fdxG_R | ACGGCAAATAAACGTCCTACGCCT | This study |
| HI_QP0_nqrB_F | TGCCAATGCAACTTGGGGCTC | [4] |
| HI_QP0_nqrB_R | ACTTCGTGACCACGAACCAC | [4] |
| HI_QP0_ackA_F | GCGAATACCAATTAAGTGAGC | [4] |
| HI_QP0_ackA_R | CGTATCGTTCACGGTGGCGA | [4] |
| Hi2019_QP_pta_F | CTGGACGATCTGCTGAAGCGACTAA | This study |
| Hi2019_QP_pta_R | TTAATGAAGGGGAAATCAATCGCCG | This study |
| HI_QP0_ldhA_F | TGATAGTTTTCTGGCGTTGC | [4] |
| HI_QP0_ldhA_R | TGAAAGGTTTTGGCATGAATAT | [4] |
| HI_QP0_lldD_F | GCAAGAATAGTTGGCATTGAAAGT | [4] |
| HI_QP0_lldD_R | GCGTAATGTGAGTGATTTGGA | [4] |
| HI_QP0_dldD_F | AAGGTGTCTAAACGAACCGCA | [4] |
| HI_QP0_dldD_R | CTATGTTCGTCAAGTTGATGAAG | [4] |
| HI_QP0_frdA_F | CAGCAAATAAACCTTTGATACG | [4] |
| HI_QP0_frdA_R | TGCTTATGAAGGTGTAAATCCA | [4] |
| HI_QP0_pflA_F | ATACTGATAGCGATCACGATGTGC | [4] |
| HI_QP0_pflA_R | CCCAAGGGTTTTCCATTTATG | [4] |
| HI_QP0_pykA_F | TTTGCTGCATACATTGCAGACAT | [4] |
| HI_QP0_pykA_R | CAGTGGCAGCAATGGCTAG | [4] |
| HI_QP0_dmsA_F | CGAACCTGATGATCAAGATTATATG | [4] |
| HI_QP0_dmsA_R | AGTAAACTGTGGTAGCCGTTG | [4] |
| HI_QP0_torZ_F | GTCGTGATTTTCTGAAAAAAACA | [4] |
| HI_QP0_torZA_R | GCAGTCACTACCGTTTTCATTTC | [4] |
| HI_QP0_msrA_F | AAAATTCAACATCATCATCTGG | [5] |
| HI_QP0_msrA_R | CCGTGAATACGCTCCATATAT | [5] |
| Hi QP cat F | CCGTCAAATTCCAGTAAACCGTCCA | [5] |
| Hi QP cat R | TGGCTGAAGCTGTTTGGCTCGTAGT | [5] |
| Hi QP pdgX F | GCTTGTGAAAAACGGCGTAGTTGAA | [5] |
| Hi QP pdgX R | TGCACTTGGTGTTGTGGTGCAAGGT | [5] |
| Hi QP sodA F | CGTTCAATCGCATCTTTTAATGCGC | [5] |
| Hi QP sodA R | TGCAGAAAAACGTGGAGCATTACGT | [5] |
| HI_Qpo_RpoE2F | AT R CCCCTTTTCATCAAAAAATGAATT | [5] |
| HI_Qpo_RpoE2R | GCATTTAA R ACTTGGATTTTTGCGATT | [5] |
| HI_Qpo_OxyRF | CGCAAAGTTTCAAG R CTAGTTGCTTGG | [5] |
| HI_Qpo_OxyRR | TGGTCAAGAAATGTTAATGCTTGATGATG | [5] |
| HI_QP0_gyrA_F | TTGGGCGTGCATTACCTGACGTT | [4] |
| HI_QP0_gyrA_R | CCCACAACACGCGCTGATTTTAC | [4] |
| HI_QP_comC_F | CTCCAGAACTGGTTGCACAAATTATTCC | This study |
| HI_QP_comC_F | CCGACTTACAGAAAGCTGTGACGCATTA | This study |
| HI_QP_comE_F | AACAGGAAGTGGCTCTTTGCTTTCTCCC | This study |
| HI_QP_comE_F | CGCCAAGTTCTTTCAAACTCTCATCCGT | This study |
| HI_QP_comF_F | GGGATAAGATGGTCATTATTGGGCATTAT | This study |
| HI_QP_comF_R | CCACTGACGAAAATGATATAAAGGCACA | This study |
| HI_QP_dprA_F | AACAAGCGGTTTAGCATTAGGAATTGATG | This study |
| HI_QP_dprA_R | TAGCAGCAATAGGTGCTTGGTTTGGTAA | This study |
| HI_QP_rec2_F | AATTTTGCACCAACAGGATTATCAAACG | This study |
| HI_QP_rec2_R | AAAAATACCATTGTTGCCGATCAAACCC | This study |

**Table S4:** Growth rates (h^-1^) of Hi2019 WT, and *ackA* and *pta* mutant strains under microaerobic and anaerobic conditions using different carbon sources. With the exception of BHI, all growth experiments used CDM medium where different main carbon sources were added to CDM base medium that additionally contains 1 mM pyruvate and 7.4 mM inosine). All data shown are average of the biological replicates data ± standard deviation.

| ***Growth condition*** | ***microaerobic*** | | | ***anaerobic*** | | |
| --- | --- | --- | --- | --- | --- | --- |
| ***Carbon Source/ Growth medium*** | **WT** | ***ΔackA*** | ***Δpta*** | **WT** | ***ΔackA*** | ***Δpta*** |
| ***sBHI*** | 0.58±0.04 | 0.34±0.07 | 0.47±0.05 | 0.48±0.01 | 0.33±0.02 | 0.34±0.05 |
| ***10mM Glucose*** | 0.62±0.01 | 0.52±0.09 | 0.39±0.07 | 0.45±0.03 | 0.27±0.03 | 0.2±0.03 |
| ***25mM Lactate*** | 0.41±0.04 | 0.22±0.03 | 0.11±0.01 | 0.53±0.01 | 0.32±0.03 | 0.19±0.04 |
| ***10mM Ribose*** | 0.56±0.02 | 0.27±0.01 | 0.12±0.02 | 0.67±0.09 | 0.33±0.00 | 0.19±0.01 |
| ***4mM Uridine*** | 0.53±0.05 | 0.37±0.01 | 0.28±0.05 | 0.45±0.04 | 0.34±0.08 | 0.30±0.01 |
| **CDMbase w pyruvate & inosine** | 0.51±0.09 | 0.22±0.06 | 0.12±0.01 | 0.57±0.01 | 0.29±0.02 | 0.26±0.07 |

**Table S5**: Metabolites produced during microaerobic and anaerobic growth of Hi2019 WT, *ackA* and *pta* mutant strains. Metabolite concentrations were determined using ^1^H-NMR, concentrations are given in mM. For each strain, three biological replicates were analyzed.

|  | ***microaerobic*** | | | ***anaerobic*** | | |
| --- | --- | --- | --- | --- | --- | --- |
| Metabolite concentrations (mM**)** | Hi2019^WT^ | Hi2019*^ΔackA^* | Hi2019*^Δpta^* | Hi2019^WT^ | Hi2019*^ΔackA^* | Hi2019*^Δpta^* |
| Glucose | 1.48±1.2 | n.d. | 0.9±0.55 | 2.15±0.29 | n.d. | 6.39±0.53 |
| Pyruvate | n.d. | n.d. | 16.7±0.91 | n.d. | n.d. | 5.65±0.21 |
| Inosine | 0.88±0.04 | 1.93±0.37 | 6.34±0.1 | 2.1±0.09 | 2.54±0.35 | 7.61±0.57 |
| Acetate | 15.11±1.01 | 11.9±0.72 | 3.39±0.09 | 10.5±0.96 | 10.2±0.70 | 1.72±0.04 |
| Hypoxanthine | 5.94±0.31 | 3.4±0.31 | 4.43±0.14 | 4.2±0.42 | 2.92±0.52 | 3.03±0.12 |
| Formate | 2.81±0.31 | 5.03±0.32 | 0.41±0.05 | 3.76±0.29 | 8.03±0.61 | 2.34±0.06 |
| Succinate | 9.21±1.02 | 3.13±0.27 | 2.82±0.18 | 8.97±1.25 | 4.64±0.31 | 3.32±0.08 |
| Lactate | 0.36±0.15 | 3.65±0.35 | 9.75±0.32 | 0.28±0.03 | 5.59±0.26 | 11.09±.49 |

**Table S6**: Phenotypic Microarray redox dye read outs – separate Excel file.

**Figure S1** – **Panel A:** *ackA* and *pta* expression in Hi2019^WT^, Hi2019^Δ^*^ackA^* and Hi2019^Δ^*^pta^* after culturing in CDM media, confirming that both mutants are non-polar. **Panel B:** Competency genes expressed in Hi2019^WT^, Hi2019^Δ^*^ackA^* and Hi2019^Δ^*^pta^* after culturing in MI-V media. Genes normalised against gyrase. Statistical analysis used two-way ANOVA with Dunnett's multiple comparisons test. **** p<0.0001, * p<0.01.

In order to ascertain that the observed effects on growth of the *ackA* mutant are related to this mutation, complementation of the mutation was attempted using a p601.1 plasmid containing a functional copy of the *pta*-*ackA* gene region, a method that has been used successfully in several previous studies [6-8]. This plasmid mediates integration of the complementation gene region into the pseudogene HI601 and was specifically designed for use with Hi2019 [3]. Despite multiple attempts, including using precultures grown on either sBHI as per our standard protocol or complete CDM, no complemented Hi2019^Δ^*^ackA^* strains were obtained. Alterations to the incubation period in competence-inducing medium (MIV) [9] and the spectinomycin concentration used for selection were also trialled and were unsuccessful. As transformation of *H. influenzae* relies on the development of competence during the incubation with the MIV medium [9], it was hypothesized that this process might be impaired in Hi2019^Δ^*^ackA^*. RNA from Hi2019^WT^ and Hi2019^Δ^*^ackA^* was isolated following incubation in MIV media which leads to the development of competence during the transformation process [9] and expression of known competence genes in both strains was determined using qPCR. This revealed that the genes required for competence, such as *comCEF*, which regulates competence, or genes which are required for DNA binding during transformation (*rec2*, *dpr*) exhibited a 2.5-fold to 6-fold reduction in expression in the mutant strain (Fig. 4). It is proposed that this reduced expression of competence genes is the reason why complementation could not be achieved, and may also have led to the failure to complement the *ackA/pta* mutation in the previous study by Lopez-Lopez, et al. [10].

**Figure S2:** Microaerobic and anaerobic growth of Hi2019^WT^ and Hi2019^Δ^*^ackA^* and Hi2019^Δ^*^pta^* on alternative main carbon sources. Data points shown are averages of three biological replicate growth curves with errors shown as standard deviations of the mean.

**Figure S3** – **Panel** **A:** Enzymatic metabolite confirmation in spent *H. influenzae* growth medium. **Pane**l **B:** qRT-PCR of genes involved in glucose (left) and fatty acid (right) metabolism. **Panel** **C:** Growth of *H. influenzae* on CDM medium supplemented with different carbon sources (in addition to 1 mM pyruvate and 7.4 mM inosine contained in the medium) that showed intense dye formation in Phenotypic Microarray tests. Substrates in bold and marked with an asterisk also showed intense dye formation in uninoculated Phenotypic Microarray plates using Biolog redox dye G (for use with fastidious bacteria). Statistical analyses used 1-Way ANOVA (panel A) or 2-Way ANOVA (Panel B) with Dunnett’s multi-comparison test. **** - p<0.0001, ** - p<0.01, * - p<0.05.

**Figure S4** Nitrogen and phosphorous sources and biofilm formation in *H. influenzae* wt and *ackA* and *pta* mutant strains. **Panel A:** Nitrogen sources used by *H. influenzae* wt and *ackA* and *pta* mutant strains as determined by Phenotypic Microarray. **Panel B:** Phosphorous sources used by *H. influenzae* wt and *ackA* and *pta* mutant strains as determined by Phenotypic Microarray.

**Figure S5** –Stress responses in Hi2019^WT^ and Hi2019*^ΔackA^* and Hi2019*^Δpta^* determined by Phenotypic Microarray plates PM09 & PM10.

**Figure S6** – Gene expression of oxidative stress gene regulators and biofilm formation **Panel A:** Expression of *oxyR* and *rpoE2* genes Hi2019 wildtype and the *ackA* and *pta* mutant strains under microaerobic conditions. Data are shown as averages with standard deviation. Statistical analyses used 2-Way ANOVA with Tukey’ multi-comparison correction. **** - p<0.0001, *** - p<0.001, **- p<0.01, *-p<0.05 **Panel B:** Biofilm stained using crystal violet, **Panel C**: Viable bacterial (CFU/ml) in biofilms. Statistical testing used 1-Way ANOVA with Dunnett’s multi-comparison correction. **** - p <0.0001, *** - p<0.001.

**Figure S7** Bacterial cell numbers during infection of 16HBE 14 tissue cells (A, B) and NHNE (C) **Panel A:** CFU/mL in inoculum for 16 HBE14 infections (all comparisons not significant) **Panel B:** planktonic bacteria (CFU/ml) during 16HBE14 infections; **Panel C:** viable bacteria (CFU/mL) in basal medium during NHNE infections with *H. influenzae* 2019 WT and mutant strains (all comparisons not significant). **Panel D:** Cell attachment assay using Murine Bone marrow-derived macrophages. Hi2019 and the *ackA* and *pta* mutant strains were incubated with macrophages for 30 minutes, after which bacterial CFU attached to macrophages were determined. inoc.- inoculum. Statistical analyses used 1-Way (Panel A) and 2-Way ANOVA (Panels B-D) with Dunnett’s multi-comparison correction. **** - p<0.0001, ** - p<0.01, comparisons that are not significant are not shown. Icons created in BioRender.com.


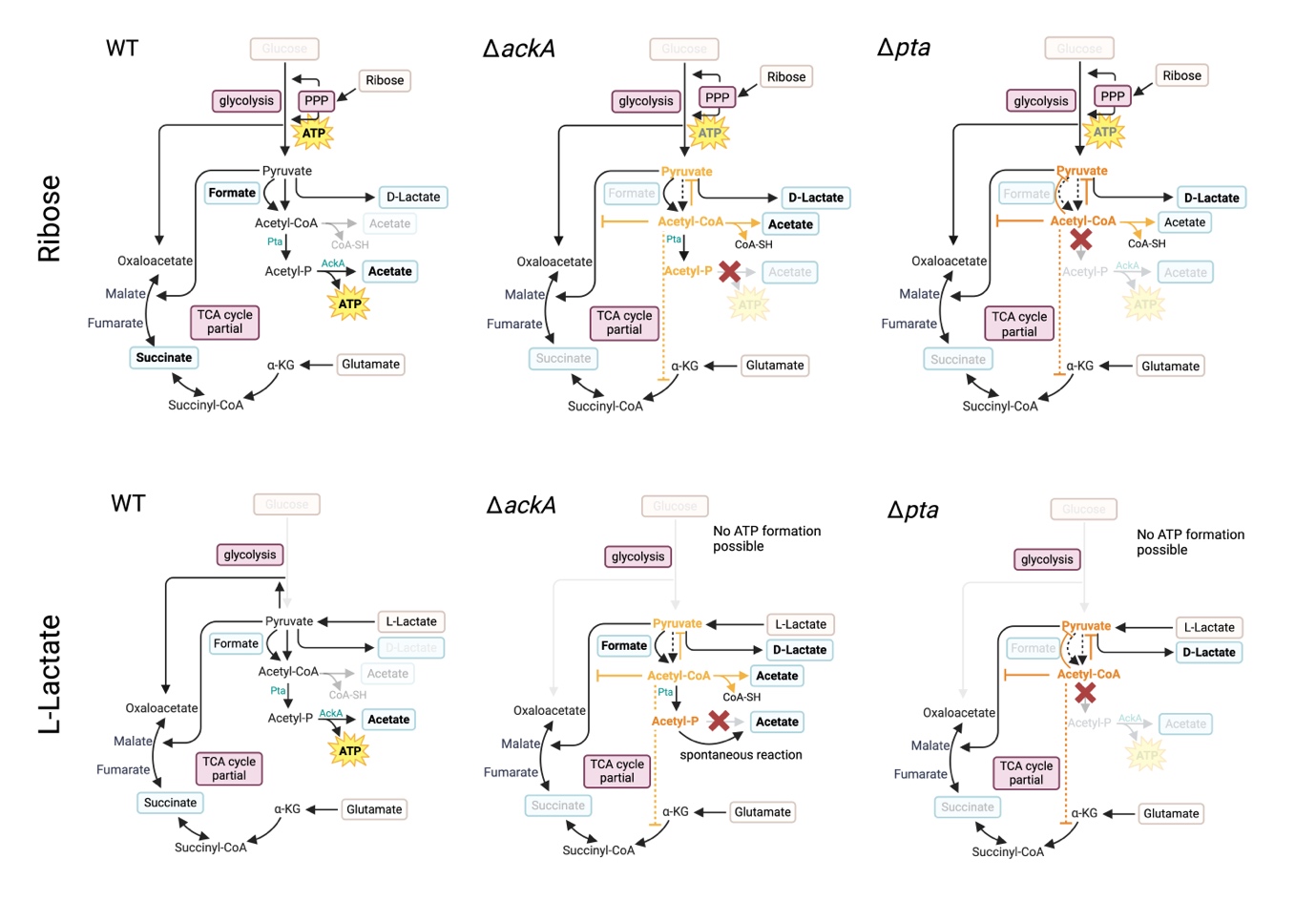


**Figure S8:** Schematic representation of the metabolic changes resulting from the Δ*ackA* and Δ*pta* mutations in *H. influenzae* during growth on ribose (top row) or L-lactate (bottom row)-containing CDM medium under anaerobic conditions. *Yellow boxes* – substrates*, purple boxes* – pathway names, *blue boxes* – metabolic endproducts, *yellow or orange metabolite names* – accumulating metabolites, *coloured arrows* – proposed reactions, black arrows –reactions with confirmed enzymes present in *H. influenzae*, *partially transparent arrows or labels*- inactive reactions or reactions with very low flux. Created in BioRender. Kappler, U. (2026) https://BioRender.com/3gcmxhu

**References – supplementary material**

1. Campagnari AA, Gupta MR, Dudas KC, Murphy TF, Apicella MA. Antigenic diversity of lipooligosaccharides of nontypable *Haemophilus influenzae*. Infect Immun. 1987;55(4):882-7; doi: 10.1128/iai.55.4.882-887.1987.

2. Vieira J, Messing J. The pUC plasmids, an M13mp7-derived system for insertion mutagenesis and sequencing with synthetic universal primers. Gene. 1982;19(3):259-68.

3. Johnston JW, Zaleski A, Allen S, Mootz JM, Armbruster D, Gibson BW, et al. Regulation of sialic acid transport and catabolism in *Haemophilus influenzae*. Mol Microbiol. 2007;66(1):26-39; doi: 10.1111/j.1365-2958.2007.05890.x.

4. Muda NM, Nasreen M, Dhouib R, Hosmer J, Hill J, Mahawar M, et al. Metabolic analyses reveal common adaptations in two invasive *Haemophilus influenzae* strains. Pathog Dis. 2019;77(2); doi: 10.1093/femspd/ftz015.

5. Nasreen M, Dhouib R, Hosmer J, Wijesinghe HGS, Fletcher A, Mahawar M, et al. Peptide methionine sulfoxide reductase from *Haemophilus influenzae* is required for protection against HOCl and affects the host response to infection. ACS Infectious Diseases. 2020;6(7):1928-39; doi: 10.1021/acsinfecdis.0c00242.

6. Hosmer J, Nasreen M, Dhouib R, Essilfie AT, Schirra HJ, Henningham A, et al. Access to highly specialized growth substrates and production of epithelial immunomodulatory metabolites determine survival of *Haemophilus influenzae* in human airway epithelial cells. PLoS Pathog. 2022;18(1):e1010209; doi: 10.1371/journal.ppat.1010209.

7. Johnston JW, Coussens NP, Allen S, Houtman JC, Turner KH, Zaleski A, et al. Characterization of the N-acetyl-5-neuraminic acid-binding site of the extracytoplasmic solute receptor (SiaP) of nontypeable *Haemophilus influenzae* strain 2019. Journal of Biological Chemistry. 2008;283(2):855-65.

8. Steele KH, O'Connor LH, Burpo N, Kohler K, Johnston JW. Characterization of a ferrous iron-responsive two-component system in nontypeable *Haemophilus influenzae*. J Bacteriol. 2012;194(22):6162-73; doi: 10.1128/JB.01465-12.

9. Poje G, Redfield RJ. Transformation of *Haemophilus influenzae*. Methods Mol Med. 2003;71:57-70; doi: 10.1385/1-59259-321-6:57.

10. Lopez-Lopez N, Euba B, Hill J, Dhouib R, Caballero LA, Leiva J, et al. *Haemophilus influenzae* Glucose Catabolism Leading to Production of the Immunometabolite Acetate Has a Key Contribution to the Host Airway-Pathogen Interplay. ACS Infect Dis. 2020;6(3):406-21; doi: 10.1021/acsinfecdis.9b00359.
